# Early aspirin withdrawal versus dual antiplatelet therapy in high-risk patients after percutaneous coronary intervention: Meta-analysis of randomized trials

**DOI:** 10.1371/journal.pmed.1004995

**Published:** 2026-03-26

**Authors:** Eliano P. Navarese, Paul Gurbel, Udaya Tantry, Giuseppe Talanas, Klaudyna Grzelakowska, Julia Umińska, Young-Hoon Jeong, Kevin Bliden, Safi U. Khan, Jacek Kubica, Timothy D. Henry, Michael E. Farkouh, Dean J. Kereiakes

**Affiliations:** 1 Department of Life and Health Sciences, Link Campus University, Rome, Italy; 2 SIRIO MEDICINE Research Network, Department of Cardiology, Nicolaus Copernicus University, Bydgoszcz, Poland; 3 Sinai Center for Thrombosis Research and Drug Development, Sinai Hospital of Baltimore, Baltimore, Maryland, United States of America; 4 Clinical Experimental Cardiology, University of Sassari, Sassari, Italy; 5 Department of Cardiology, Nicolaus Copernicus University, Bydgoszcz, Poland; 6 Division of Cardiology, Department of Internal Medicine, Chung-Ang University College of Medicine, Seoul, South Korea; 7 CAU Thrombosis and Biomarker Center, Chung-Ang University Gwangmyeong Hospital, Gwangmyeong, South Korea; 8 Department of Interventional Cardiology, Baylor Scott and White Heart Hospital, Plano, Texas, United States of America; 9 The Carl and Edyth Lindner Center for Research and Education, Cincinnati, Ohio, United States of America; 10 The Christ Hospital, Cincinnati, Ohio, United States of America; 11 Departments of Academic Affairs and Cardiology, Cedars-Sinai Health System, Los Angeles, California, United States of America; Peking University, CHINA

## Abstract

**Background:**

Patients at high ischemic or bleeding risk after percutaneous coronary intervention (PCI) require protection against thrombotic events with dual antiplatelet therapy (DAPT) while avoiding bleeding. Although guidelines recommend 12-month DAPT after acute coronary syndrome (ACS), recent trials have tested the safety of early aspirin withdrawal with potent P2Y12-inhibitor monotherapy.

**Methods and findings:**

We performed a meta-analysis of randomized trials (from inception through August 2025) comparing early aspirin withdrawal (≤3 months) with transition to ticagrelor- or prasugrel-monotherapy versus continued DAPT. Co-primary outcomes were myocardial infarction (MI) and clinically relevant bleeding. Prespecified timing analyses stratified the comparison versus DAPT by aspirin timing: immediate (aspirin noninitiation or in-hospital cessation) and early (post-discharge discontinuation within 3 months). Bayesian models quantified risk-stratified probabilities of benefit and harm; trial sequential analysis (TSA) assessed conclusiveness of evidence. Seven trials (*n* = 27,743) were included. P2Y12-inhibitor monotherapy reduced bleeding (HR = 0.55, 95% CI [0.42, 0.71]; *p* < 0.001) without significantly increasing MI overall (HR = 1.11, 95% CI [0.91, 1.35]; *p* = 0.31), death, stroke, or stent thrombosis. Immediate aspirin noninitiation/cessation increased MI (HR = 1.41, 95% CI [1.01, 1.97]; *p* = 0.04), whereas early discontinuation did not (HR = 0.97, 95% CI [0.76, 1.24]; *p* = 0.82). TSA indicated conclusiveness for bleeding benefit and futility for an MI excess. Analyses restricted to ACS confirmed the overall results. Bayesian analyses corroborated these effects and identified risk-aligned timing: in high bleeding risk, ≤1-month aspirin discontinuation yielded a 100% posterior probability of bleeding benefit (NNT = 12) and 70% probability of MI-safety; in high ischemic risk, 3-month aspirin discontinuation yielded 100% probability of bleeding benefit (NNT = 57) and 86% probability of MI-safety. Limitations include aggregate data only and limited precision for the immediate aspirin withdrawal subgroup.

**Conclusions:**

Among high-risk post-PCI patients on ticagrelor/prasugrel, discontinuing aspirin within 3 months reduces bleeding without an ischemic trade-off versus DAPT. Immediate aspirin noninitiation or cessation should be avoided; timing should be individualized to bleeding and ischemic risk. PROSPERO: CRD420251167706.

## Author summary

**Why was this study done?:**

- After a coronary stent procedure, patients take two antiplatelet medicines to reduce the risk of ischemic events, but this can increase bleeding.
- Many patients are at higher risk of ischemic events or bleeding than lower-risk patients after percutaneous coronary intervention, so doctors need safer ways to balance these two risks. Several trials have tested stopping aspirin early and continuing only the other antiplatelet medicine, but the safest timing—especially in high-risk patients—has been uncertain.

**What did the researchers do and find?:**

- We performed a meta-analysis of 7 randomized clinical trials including 27,743 high-risk patients after a stent procedure.
- Overall, stopping aspirin within 3 months and continuing a potent antiplatelet medicine reduced bleeding compared with staying on two medicines, without a clear increase in myocardial infarction (MI), death, or stroke.
- However, not starting aspirin or stopping it immediately in hospital was linked to more MI than continuing two medicines.

**What do these findings mean?:**

- For many high-risk patients after a stent procedure, stopping aspirin within the first 1–3 months while continuing the other antiplatelet medicine may lower bleeding risk without increasing MI overall.
- Stopping aspirin right away may be risky and should generally be avoided; decisions about timing should be made with the treating clinician based on the patient’s bleeding and ischemic risk.
- A limitation of this study is that our conclusions are based on summary results reported in published trials, not individual-patient data, and only a few studies tested stopping aspirin immediately, so those specific results should be considered hypothesis-generating.

## Abstract 3

In a meta-analysis, Eliano Navarese and colleagues investigate the effect of early aspirin withdrawal after a percutaneous coronary intervention on risk of myocardial infarction and bleeding using data from randomized trials

## Introduction

Dual antiplatelet therapy (DAPT) with aspirin plus a P2Y12 inhibitor has long been the antithrombotic cornerstone after percutaneous coronary intervention (PCI) with distinct durations compared over time [1]. In contemporary practice, many patients are at high ischemic or bleeding risk, requiring a careful balance between thrombotic protection and bleeding harm. Current guidelines generally default to 12-month DAPT with aspirin and preferably a potent P2Y12 inhibitor (ticagrelor/prasugrel) after acute coronary syndrome (ACS), with shorter or longer durations reserved for selected patients according to ischemic and bleeding risk [2]. Clinical decision-making, therefore, requires navigating a fundamental trade-off: maximizing protection from thrombotic events while minimizing bleeding harm. In this context, strategies withdrawing aspirin at 3 months generally reduced bleeding without excess ischemic events [3,4], whereas earlier studies have yielded heterogeneous findings [5,6]. These observations suggest that timing of aspirin withdrawal may be a key determinant of net clinical effect with antithrombotic treatments. However, the timing of aspirin cessation varies across studies, and available trials are underpowered for definitive conclusions on individual endpoints.

To address this uncertainty, we conducted a meta-analysis of randomized trials in high-risk post-PCI populations, applying both pairwise random-effects and Bayesian models. We compared, against continued DAPT, a strategy of aspirin withdrawal within 3 months followed by ticagrelor/prasugrel monotherapy. We then assessed whether timing—immediate (aspirin noninitiation or in-hospital cessation) versus early (post-discharge discontinuation within 3 months)—modified bleeding and ischemic outcomes.

## Methods

Established methods recommended by the Cochrane Collaboration and the Preferred Reporting Items for Systematic reviews and Meta-Analyses (PRISMA) statement were used [7,8]. A systematic literature search of PubMed, Embase, and the Cochrane Central Register of Controlled Trials was conducted from inception through August 2025. The meta-analysis is registered in PROSPERO (CRD420251167706); the study protocol is publicly available through the PROSPERO database and can be accessed via the PROSPERO registry. This study is reported as per the PRISMA guideline (S1 PRISMA Checklist).

### Search strategy and selection process

The electronic search strategy used keywords related to aspirin discontinuation/aspirin-free strategies, P2Y12 inhibitor monotherapy, DAPT, and PCI. No language restrictions were applied. Reference lists of relevant trials and review articles were manually screened to identify additional eligible studies.

Eligibility criteria were randomized, parallel-group trials of adults undergoing PCI that compared potent P2Y12-inhibitor monotherapy (ticagrelor or prasugrel) after aspirin noninitiation or cessation within 3 months versus continued DAPT. Populations had to be at high ischemic or bleeding risk, defined as ACS at presentation and/or protocol-specified risk features (≥1 clinical plus ≥1 angiographic high-risk feature), or trial-defined high bleeding risk per consensus criteria (Table 1). To preserve randomization, isolate the effect of aspirin withdrawal, and enable unbiased pooling across clinically comparable high-risk post-PCI trials, as formally recommended [7], we prespecified the following exclusion criteria: (1) populations not restricted by design to high-risk post-PCI patients (or only post-hoc high-risk subgroups, which break randomization); (2) the experimental regimen was clopidogrel monotherapy or mandated early switch/de-escalation to clopidogrel; (3) the design was nonrandomized, crossover, or otherwise nonparallel-group, or outcomes were available only from post-randomization subgroups that break randomization; (4) aspirin withdrawal could not be isolated because a co-intervention was mandated at the time aspirin was stopped (e.g., P2Y12-inhibitor switch or dose reduction); (5) treatment regimens diverged after 12 months so that the comparison no longer reflected potent P2Y12-inhibitor monotherapy versus continued DAPT; or (6) the trial, by design, employed polymer-free drug-coated stents rather than conventional drug eluting stents (DES) and incorporated planned P2Y12 de-escalation, thereby precluding isolation of the aspirin withdrawal effect.

**Table 1:** Baseline characteristics of included trials.

Study	Regimen comparison	P2Y12 inhibitor type	Time of aspirin withdrawal	Number of participants			Age, years	Female sex, %	ACS type	High-risk features	Follow-up period
**Total**	**P2Y12 mono**	**DAPT**					
NEO-MINDSET	In-hospital DAPT followed by potent P2Y12 inhibitor alone vs. 12-month DAPT	ticagrelor 28.8%, prasugrel 69.3%	In hospital	3,410	1,712	1,698	59.6	29.3	STEMI 62.1%, NSTEMI 30.5%, UA 7.4%	ACS	12 months
STOPDAPT-3	Immediate prasugrel monotherapy vs. 1-month DAPT	prasugrel	No aspirin after randomization (immediate)	5,966	2,984	2,982	71.6	23.4	ACS 75% (STEMI 57.0%, NSTEMI 24.5%, UA 18.5%), non-ACS 25%	Inclusion criterion: non-ACS with high bleeding risk defined by the Academic Research Consortium criteria or ACS	1 month
TARGET-FIRST	1-month DAPT followed by P2Y12 inhibitor alone vs. 12- month DAPT	ticagrelor 74.0%, prasugrel 20.9%, clopidogrel 5.1%	1 month	1942	961	981	61.0	21.6	STEMI 50.4%, NSTEMI 49.6%	ACS	12 months
TICO	3-month DAPT followed by ticagrelor alone vs. 12-month DAPT	ticagrelor	3 months	3,056	1,527	1,529	61	20.5	STEMI 36.1%, NSTEMI 33.6%, UA 30.3%	ACS	12 months
T-PASS	≤1-month DAPT followed by ticagrelor alone vs. 12- month DAPT	ticagrelor	≤1 month	2,850	1,426	1,424	61	16.7	STEMI 40.4%, NSTEMI 34.8%, UA 24.8%	ACS	12 months
TWILIGHT	3-month DAPT followed by ticagrelor alone vs. 12- month DAPT	ticagrelor	3 months	7,119	3,555	3,564	65	23.8	NSTEMI 45.9%, UA 54.1%	inclusion criterion (high ischemic or bleeding risk): at least one clinical (age ≥ 65 years, female sex, troponin positive ACS, atherosclerotic vascular disease, diabetes requiring medication, or chronic kidney disease) and one angiographic feature associated with a high-risk of ischemic or bleeding events (multivessel coronary- artery disease, total stent length > 30 mm, thrombotic target lesion, bifurcation lesion requiring two stents, obstructive left main or proximal left anterior descending lesion, or calcified target lesion requiring atherec- tomy)	12 months
ULTIMATE-DAPT	1-month DAPT followed by ticagrelor alone vs. 12- month DAPT	ticagrelor	1 month	3,400	1,700	1,700	63	25.6	STEMI 27.9%, NSTEMI 31.6%, UA 40.5%	ACS	12 months

NEO-MINDSET = Early Withdrawal of Aspirin after PCI in Acute Coronary Syndromes; STOPDAPT-3 = An Aspirin-Free versus Dual Antiplatelet Strategy for Coronary Stenting: STOPDAPT-3 Randomized Trial; TARGE-FIRST = Early Discontinuation of Aspirin after PCI in Low-Risk Acute Myocardial Infarction; TICO = Effect of Ticagrelor Monotherapy versus Ticagrelor With Aspirin on Major Bleeding and Cardiovascular Events inPatients With Acute Coronary Syndrome (TICO); T-PASS = Stopping Aspirin Within 1 Month After Stenting for Ticagrelor Monotherapy in Acute Coronary Syndrome; TWILIGHT = Ticagrelor with or without Aspirin in High-Risk Patients after PCI; ULTIMATE-DAPT = Ticagrelor alone versus ticagrelor plus aspirin from month 1 to month 12 after percutaneous coronary intervention in patients with acute coronary syndromes (ULTIMATE-DAPT).

ACS = acute coronary syndrome; DAPT = dual antiplatelet therapy; STEMI = ST-elevation myocardial infarction; NSTEMI = non-ST elevation myocardial infarction; UA = unstable angina.

Two investigators (KG and JU) independently screened all titles and abstracts identified from the literature search to assess eligibility. Full-text articles were then independently reviewed for inclusion according to prespecified criteria. Discrepancies were resolved through discussion and consensus, with adjudication by a third investigator (EPN) when necessary. No automation tools were used in the study selection process.

### Data collection process

Data extraction was independently performed by two investigators (KG and JU) using a standardized, predefined data collection form. Extracted variables included study design, population characteristics, intervention details, outcome definitions, and effect estimates. Any discrepancies were resolved by consensus. When necessary, original publications and supplementary materials were reviewed to confirm accuracy. No automation tools were used.

We prespecified MI and clinically relevant bleeding as co-primary endpoints to capture the core efficacy–safety trade-off of antiplatelet therapy after PCI in these high-risk patients. Secondary outcomes were major bleeding, all-cause death, cardiovascular death, stroke, and stent thrombosis. Composite MACE endpoints were considered less informative for this question because they can mask opposite effects on ischemic versus bleeding outcomes [1,9,10]. Clinically relevant bleeding was defined as Bleeding Academic Research Consortium (BARC) types 2–5, and major bleeding as BARC types 3–5, where available. All included trials employed Myocardial Infarction (MI) definitions based on either the Universal Definition of Myocardial Infarction (Third or Fourth edition) or the Academic Research Consortium (ARC) criteria, which are aligned with Universal Definition principles. All trials included periprocedural MI as part of the MI endpoint, and all events were adjudicated by independent Clinical Endpoint Committees using predefined criteria (Table A in S1 Appendix).

### Timing definitions and allocation

Prespecified timing analyses stratified the effect of P2Y12 monotherapy versus DAPT by aspirin timing: immediate (aspirin noninitiation or cessation during the index hospitalization) and early (post-discharge discontinuation within 3 months). Within the early window, we further prespecified two strata by trial time point: ≤1 month and 3 months.

### Statistical analysis

Trial-level data were analyzed according to the intention-to-treat principle. Hazard ratios (HRs) with 95% confidence intervals (CIs) were abstracted from individual studies. Pairwise meta-analyses used a conservative random-effects model with the DerSimonian–Laird estimator for between-study variance (τ²) as the primary approach [11], with heterogeneity summarized by I² for each outcome [12]. As a sensitivity analysis, we reanalyzed all endpoints using alternative random-effects models based on the Paule–Mandel estimator with Hartung–Knapp adjustment. We also performed sensitivity analyses restricted to ACS presentations and leave-one-out analyses omitting each trial in turn to assess the influence of individual studies on pooled estimates. Prespecified aspirin withdrawal timing strata—immediate and early (≤3 months)—were analyzed using separate random-effects models. Small-study effects were explored with funnel plots [13].

We performed trial sequential analysis (TSA) to assess evidential conclusiveness, calculating the diversity-adjusted required information size and constructing O’Brien–Fleming–type monitoring boundaries (two-sided α = 0.05; power 80%–90%), classifying cumulative evidence as efficacy, futility, or inconclusive according to boundary crossings [14].

To address residual uncertainty and to provide probabilistic inferences, we additionally fitted Bayesian random-effects hierarchical models on the log–hazard-ratio scale [15]. For each meta-analytic contrast, study-specific log-HRs (θᵢ) with known standard errors (σᵢ) were assumed to arise from a normal distribution N(θ, σᵢ² + τ²), where θ denotes the true underlying log-hazard ratio and τ² the between-trial variance. We used weakly informative priors θ ~ N(0, 0.35²) and τ ~ half-normal(0.2). Posterior draws were obtained via direct Monte Carlo sampling from the conjugate normal–normal model, and we report posterior medians, 95% credible intervals, and posterior probabilities that HR < 1 or HR > 1 as measures of directional evidence for benefit or harm. We prespecified two clinical risk profiles—high bleeding risk and high ischemic risk—and derived NNT by projecting pooled Bayesian HRs onto the quartiles of the observed control-arm baseline-risk distribution across trials. For scenario-based clinical interpretation, posterior HR samples for MI and bleeding were mapped onto absolute risks using prespecified 12-month baseline risks representative of low-MI/high-bleeding and high-MI/low-bleeding profiles. We then derived joint posterior probabilities for each strategy, achieving a reduction in clinically relevant bleeding while keeping any relative increase in MI within predefined clinically acceptable bounds. As a numerical and prior-sensitivity diagnostic, we examined the stability of the Bayesian random-effects results with respect to Monte Carlo error and to the prior distribution on τ. MI noninferiority was prespecified with a margin of HR 1.15. This threshold preserves ≈50% of the established proportional benefit of aspirin versus standard care on serious vascular events among high-risk patients, as reported by the Antithrombotic Trialists’ Collaboration [16]. We also performed sensitivity analyses using a wider noninferiority margin (HR 1.30) commonly applied in antithrombotic trials [17]. All analyses were conducted in Python and R; frequentist tests used two-sided α = 0.05.

## Results

### Study selection and characteristics

Seven randomized trials (*n* = 27,743) enrolling high-risk patients undergoing PCI with potent P2Y12 inhibitors met inclusion criteria [3,4,6,17–19] (Fig A in S1 Appendix). For the ACS-restricted sensitivity analysis, we pooled either ACS-only trials or the ACS subgroup [20]. ACS was the predominant presentation and potent P2Y12-inhibitor monotherapy (ticagrelor or prasugrel) was uniformly used (Table 1) with only minimal clopidogrel use in one trial, ~5% [19]. Two trials tested immediate aspirin noninitiation or cessation; the remaining studies discontinued aspirin at ≤1–3 months, with 12-month follow-up in most. Owing to a protocol-specified antiplatelet changes at 1 month, analyses of STOPDAPT-3 were restricted to 30-day endpoints, aligning with the primary analysis of the trial. The sample-size–weighted mean age across trials was 64.2 years (trial-level mean ages ranged from 59.6 to 71.6; Table 1).

At the study level, risk of bias was appraised with the Cochrane RoB 2 tool across the domains of randomization (D1), deviations from intended interventions (D2), missing outcome data (D3), outcome measurement (D4), and selective reporting (D5). The randomization process was judged low-risk in all trials (Table B in S1 Appendix). The certainty of evidence (GRADE) for each outcome is summarized in Table C in S1 Appendix. Visual inspection of funnel plots suggested no publication bias for the investigated outcomes (Fig B in S1 Appendix). The open-label trials [3,6,17–19] were generally rated some concerns for D2 and D4 due to the potential for performance and detection bias in the knowledge of treatment assignment, although missing outcome data were low and major endpoints were largely objective; selective reporting was not identified as a major concern.

### Clinical outcomes

#### Myocardial infarction.

**Fig 1:**
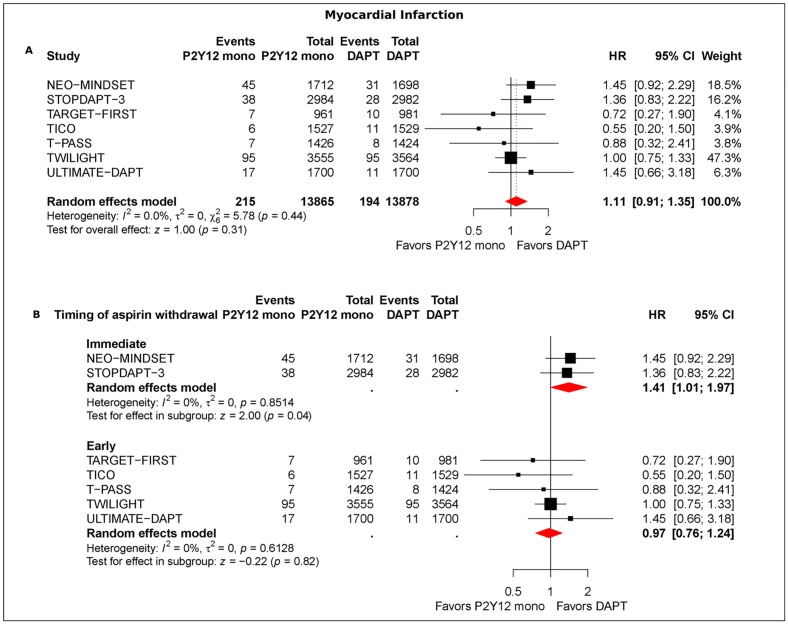
Myocardial infarction (MI) with potent P2Y12-inhibitor monotherapy vs. dual antiplatelet therapy (DAPT) after percutaneous coronary intervention (PCI). Forest plot of hazard ratios (HRs) with 95% confidence intervals. Pooled estimates were obtained using a random-effects model. *P* values for the overall pooled effect (and for individual studies, where shown) are from a two-sided *z* (Wald) test. **Panel A:** Overall random-effects meta-analysis of MI comparing P2Y12-inhibitor monotherapy (ticagrelor or prasugrel) after early after aspirin withdrawal within 3 months vs. continued DAPT. **Panel B:** Prespecified timing analysis stratifying effects on MI vs. DAPT by aspirin timing—Immediate (in-hospital aspirin noninitiation or cessation) and Early (post-discharge discontinuation within 3 months). Point estimates are hazard ratios (HRs) with 95% confidence intervals (CIs); symbol sizes are proportional to inverse-variance weights; diamonds denote pooled effects. NEO-MINDSET = Early Withdrawal of Aspirin after PCI in Acute Coronary Syndromes; STOPDAPT-3 = An Aspirin-Free vs. Dual Antiplatelet Strategy for Coronary Stenting: STOPDAPT-3 Randomized Trial; TARGET-FIRST = Early Discontinuation of Aspirin after PCI in Low-Risk Acute Myocardial Infarction; TICO = Effect of Ticagrelor Monotherapy vs. Ticagrelor With Aspirin on Major Bleeding and Cardiovascular Events inPatients With Acute Coronary Syndrome (TICO); T-PASS = Stopping Aspirin Within 1 Month After Stenting for Ticagrelor Monotherapy in Acute Coronary Syndrome; TWILIGHT = Ticagrelor with or without Aspirin in High-Risk Patients after PCI; ULTIMATE-DAPT = Ticagrelor alone vs. ticagrelor plus aspirin from month 1 to month 12 after percutaneous coronary intervention in patients with acute coronary syndromes (ULTIMATE-DAPT).

MI data were available from 7 trials (*n* = 27,743). Pooled event rates were 1.55% (215/13,865) with potent P2Y12 monotherapy and 1.39% (194/13,878) with DAPT. MI did not differ significantly between strategies (HR 1.11; 95% CI [0.91, 1.35]; *p* = 0.31; I2 = 0%) (Fig 1A). By timing, immediate aspirin noninitiation or cessation was associated with higher MI risk versus DAPT (HR 1.41; 95% CI [1.01, 1.97]; *p* = 0.04; I2 = 0%), whereas early discontinuation was not (HR 0.97; 95% CI [0.76, 1.24]; *p* = 0.82; I2 = 0%) (Fig 1B). Within the early window, estimates were HR 1.04 (95% CI [0.61, 1.75]; *p* = 0.89) for ≤1 month and HR 0.91 (95% CI [0.59, 1.40]; *p* = 0.65) for 3 months with no significant between-strategy difference (Fig C in S1 Appendix). Sensitivity analysis restricted to ACS patients confirmed overall neutrality (HR 1.07; 95% CI [0.85, 1.35]; *p* = 0.55) (Fig D in S1 Appendix).

### Bleeding

**Fig 2:**
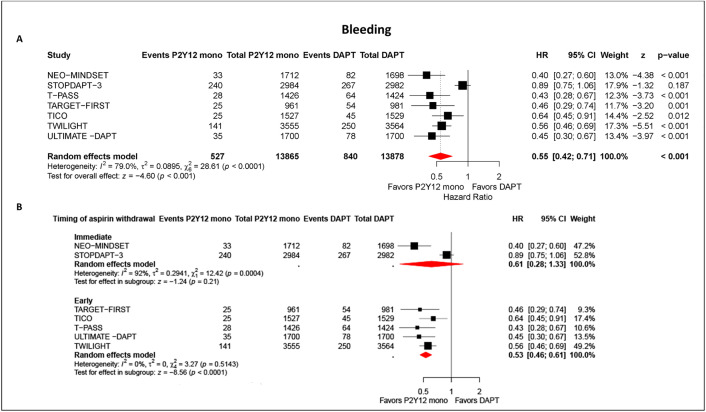
Bleeding with potent P2Y12-inhibitor monotherapy vs. 12-month dual antiplatelet therapy (DAPT) after percutaneous coronary intervention (PCI). Point estimates are hazard ratios (HRs) with 95% confidence intervals (CIs); symbol sizes are proportional to inverse-variance weights; diamonds denote pooled effects. Pooled estimates were obtained using a random-effects model. *P* values for the overall pooled effect are from a two-sided *z* (Wald) test. **Panel A**: Overall random-effects meta-analysis of bleeding comparing P2Y12-inhibitor monotherapy (ticagrelor or prasugrel) after early after aspirin withdrawal within 3 months vs. continued DAPT. **Panel B:** Prespecified timing analysis stratifying effects on bleeding vs. DAPT by aspirin timing—Immediate (in-hospital aspirin noninitiation or cessation) and Early (post-discharge discontinuation within 3 months). Point estimates are hazard ratios (HRs) with 95% confidence intervals (CIs); symbol sizes reflect inverse-variance weight; diamonds indicate pooled effects. Pooled estimates were obtained using a random-effects model. *P* values for the overall pooled effect are from a two-sided z (Wald) test. Immediate = noninitiation or cessation ≤4 days; early = discontinuation >4 days to ≤3 months. Remaining abbreviations as in Fig 1.

Data from 7 trials (*n* = 27,743) showed 527/13,865 bleeding events (3.80%) with potent P2Y12 monotherapy versus 840/13,878 (6.05%) with DAPT, a highly significant difference (HR 0.55, 95% CI [0.42–0.71]; *p* < 0.001; I2 = 79.0%) (Fig 2A). In timing analyses (Fig 2B), immediate aspirin noninitiation or cessation yielded a nonsignificant lower bleeding risk versus DAPT (HR 0.61, 95% CI [0.28, 1.33]; *p* = 0.21 I² = 92%), whereas early discontinuation significantly reduced bleeding (HR 0.53, 95% CI [0.46, 0.61]; *p* < 0.001; I² = 0%). Within the early window, stratum-specific pooled effects were similar: ≤1 month (HR 0.51, 95% CI [0.34, 0.77]; *p* = 0.001) and 3 months (HR 0.58, 95% CI 0.49, 0.69; *p* < 0.001) (Fig E in S1 Appendix). A sensitivity analysis restricted to ACS patients confirmed a significant bleeding reduction with P2Y12 monotherapy (HR 0.48, 95% CI [0.40, 0.58]; *p* < 0.001) (Fig F in S1 Appendix).

#### Major bleeding.

Pooled rates were 1.02% (94/9,169) with monotherapy and 2.14% (197/9,198) with DAPT. Major bleeding estimates were available from trials implementing early aspirin withdrawal and showed a robust reduction with P2Y12 monotherapy (HR 0.48, 95% CI [0.38, 0.62]; *p* < 0.001) (Fig G in S1 Appendix).

### Trial sequential analysis

#### TSA.

**Fig 3:**
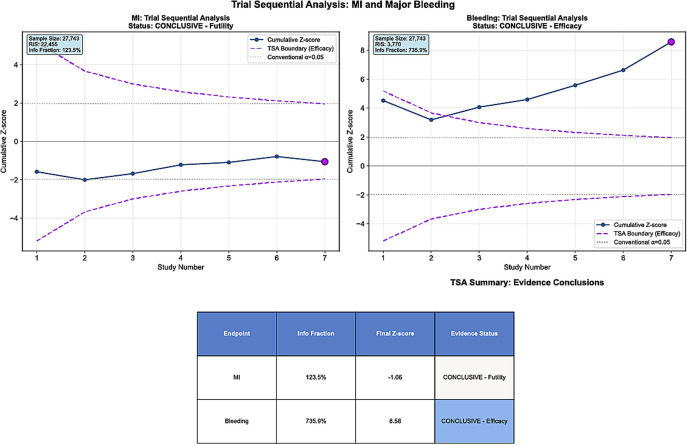
Trial sequential analysis (TSA). For TSA, we (i) estimated the control-arm event rate from the pooled DAPT arms, (ii) calculated the diversity-adjusted required information size assuming a two-sided α = 0.05 and 80%–90% power, and (iii) constructed O’Brien–Fleming–type monitoring boundaries. Right **Panel (Bleeding):** The cumulative z-curve for study-defined bleeding crosses the O’Brien–Fleming efficacy boundary and reaches/exceeds the diversity-adjusted required information size (RIS), indicating conclusive evidence favoring P2Y₁₂-inhibitor monotherapy. Left **Panel (Myocardial infarction):** The cumulative z-curve remains within the futility region for a clinically important difference in MI between strategies. Abbreviations: MI = myocardial infarction; RIS = required information size; TSA = trial sequential analysis. DAPT = dual antiplatelet therapy.

For study-defined bleeding, the cumulative evidence reached and exceeded the required information size (RIS)—the amount of information that would be needed for a single, adequately powered randomized trial. The cumulative curve crossed the efficacy boundary, indicating that the bleeding reduction with potent P2Y12 monotherapy is supported by conclusive evidence, and that additional trials are unlikely to overturn this finding Fig 3, Bleeding).

For MI, even though the overall information was sufficient, the cumulative curve remained within the futility region, supporting no clinically important difference between strategies at the population level (Fig 3, MI).

### Bayesian meta-analysis

For MI, the posterior median HR was 1.10 (95% CrI 0.89–1.35) with low heterogeneity (τ = 0.06), indicating a low probability of MI benefit with early aspirin discontinuation plus potent P2Y12 monotherapy. In contrast, for bleeding the posterior median HR was 0.59 (95% CrI [0.46, 0.78], with P(HR < 1); heterogeneity was moderate (τ = 0.39) (Fig H in S1 Appendix).

**Fig 4:**
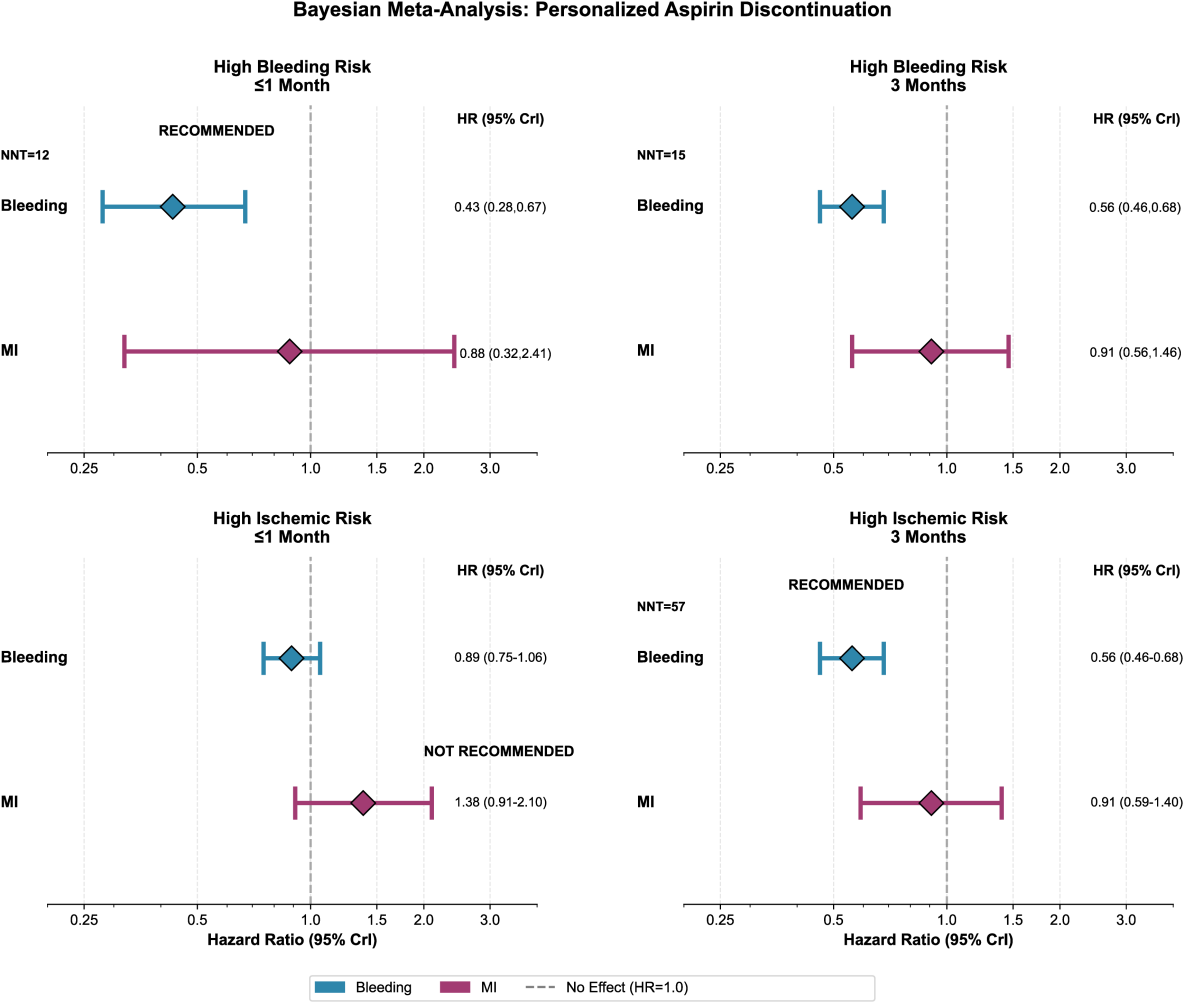
Forest plots showing Bayesian random-effects meta-analyses stratified by clinical risk profile and aspirin discontinuation timing. Top panels: high bleeding risk. Bottom panels: high ischemic risk. Left panels: ≤1-month discontinuation. Right panels: 3-month discontinuation. Each panel displays bleeding (blue diamonds) and myocardial infarction (purple diamonds) with hazard ratios (posterior medians) and 95% Bayesian credible intervals. Green badges mark recommended strategies based on posterior probabilities: ≤1 month for high bleeding risk (100% probability of bleeding benefit; number needed to treat (NNT) = 12; 70% probability that HR_MI < 1.15) and 3 months for high ischemic risk (100% probability of bleeding benefit; NNT = 57; 86% probability that HR_MI < 1.15). Red label indicates a nonrecommended strategy (high ischemic risk at ≤1 month: 20% probability that HR_MI < 1.15). The dashed line represents no effect (HR = 1.0). **Abbreviations:** NNT = number needed to treat to prevent one bleeding event; CrI = credible interval.

To inform personalized timing strategies, we conducted prespecified exploratory Bayesian analyses stratified by clinical risk profile (high bleeding risk; high ischemic risk) and quantified decision-relevant probabilities. In high bleeding risk, ≤1-month discontinuation showed a 100% posterior probability of bleeding reduction (HR 0.43, 95% CrI [0.28, 0.67]; NNT = 12) with moderate MI-safety confidence (HR 0.88, 95% CrI [0.32, 2.41]; 70% probability that the HR for MI < 1.15). In high ischemic risk, ≤ 1-month aspirin discontinuation provided limited MI-safety confidence (HR 1.38, 95% CrI [0.91, 2.10]; 20% probability that the HR for MI < 1.15), whereas 3-month aspirin discontinuation achieved both robust bleeding reduction (HR 0.56, 95% CrI [0.46, 0.68]; NNT = 57) and high MI-safety confidence (HR 0.91, 95% CrI [0.59, 1.40]; 86% probability that the HR for MI < 1.15) (Fig 4). With a more lenient MI-safety margin (HR < 1.30), results were consistent: in high bleeding risk, ≤1-month discontinuation showed 100% posterior probability of bleeding benefit and 78% probability of MI-safety; in high ischemic risk, 3-month discontinuation showed 100% probability of bleeding benefit and 95% probability of MI-safety. In Bayesian random-effects models, posterior estimates for bleeding and MI were essentially unchanged when increasing posterior draws (10,000–50,000) and when varying the prior for τ, indicating robustness to Monte Carlo error and prior choice (Table D in S1 Appendix).

### All-cause death

**Fig 5:**
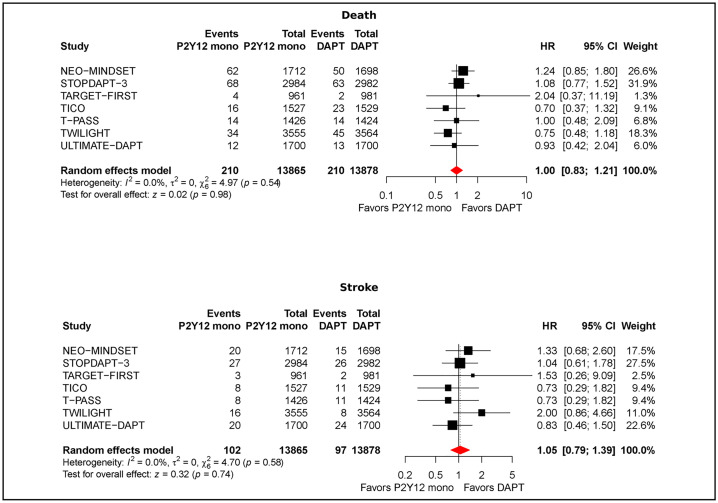
Other clinical outcomes —death and stroke. **Panel A:** Overall random-effects meta-analysis of death comparing P2Y12-inhibitor monotherapy (ticagrelor or prasugrel) after early after aspirin withdrawal within 3 months vs. continued DAPT. **Panel B:** Overall random-effects meta-analysis of stroke comparing P2Y12-inhibitor monotherapy (ticagrelor or prasugrel) after early after aspirin withdrawal within 3 months vs. continued DAPT. Remaining abbreviations as in Fig 1. Point estimates are hazard ratios (HRs) with 95% confidence intervals (CIs); symbol sizes reflect inverse-variance weight; diamonds indicate pooled effects. Pooled estimates were obtained using a random-effects model. *P* values for the overall pooled effect are from a two-sided *z* (Wald) test.

All-cause death event rates were 1.51% (210/13,865) with P2Y12 monotherapy and 1.65% (210/12,655) with DAPT. There was no difference between strategies (HR 1.00; 95% CI [0.83, 1.21]; *p* = 0.98; I² = 0%) (Fig 5A). In timing analyses (Fig I in S1 Appendix), pooled HRs were 1.15 (95% CI [0.89, 1.48]; *p* = 0.27; I² = 0%) for immediate noninitiation or cessation and 0.82 (95% CI [0.61, 1.11]; *p* = 0.20; I² = 0%) for early discontinuation, with no significant between-strategy differences. Within the early window, stratum-specific pooled effects were: ≤1 month: HR 1.03 (95% CI [0.62, 1.73]; *p* = 0.89); 3 months: HR 0.73 (95% CI [0.51, 1.06]; *p* = 0.09) (Fig J in S1 Appendix). Sensitivity analysis restricted to ACS patients confirmed no significant differences between strategies (HR 0.96, 95% CI [0.74, 1.24]; *p* = 0.74) (Fig K in S1 Appendix). Pooled estimates for cardiovascular death mirrored all-cause death, with a pooled HR near unity and confidence intervals spanning 1.0 (no between-strategy difference (Fig L in S1 Appendix).

### Stroke

Stroke pooled rates were 0.73% (102/13,865) with P2Y12 monotherapy and 0.69% (97/13,878) with DAPT. The random-effects estimate showed no difference between potent P2Y12-inhibitor monotherapy and continued DAPT (HR 1.05; 95% CI [0.79, 1.39]; *p* = 0.74; I² = 0%) (Fig 5B). In timing analyses (Fig M in S1 Appendix) there were no significant differences: pooled HRs were 1.14 (95% CI [0.75, 1.74]; *p* = 0.52) for immediate noninitiation or cessation and 0.98 (95% CI [0.66, 1.43]; *p* = 0.89) for early discontinuation. Within the early window, stratum-specific analyses were neutral in both strata (Fig N in S1 Appendix) and results were similarly neutral in ACS (Fig O in S1 Appendix). Across six randomized trials, definite/probable stent thrombosis was not significantly different between potent P2Y12-inhibitor monotherapy and DAPT (Fig P in S1 Appendix).

**Fig 6:**
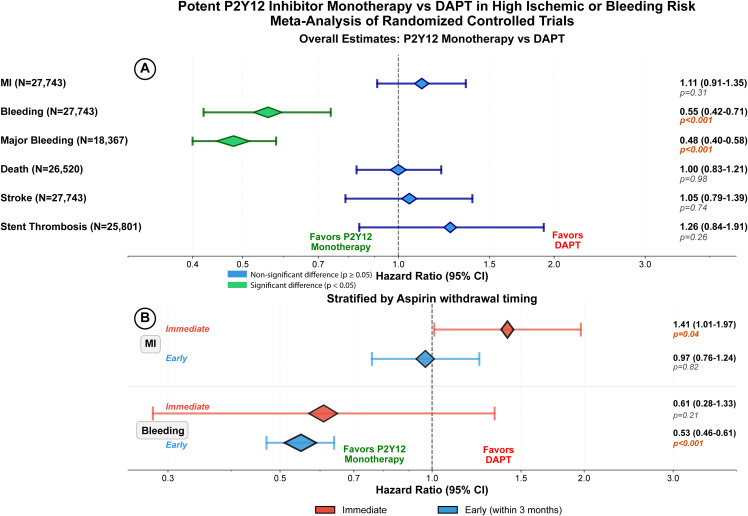
Timing-dependent effects of aspirin withdrawal. Overall estimates (Panel **A)** and timing-stratified analysis (Panel **B)**—immediate (in-hospital aspirin noninitiation or cessation) and early (post-discharge discontinuation within 3 months)—comparing P2Y12-inhibitor monotherapy (ticagrelor or prasugrel) vs. dual antiplatelet therapy (DAPT) in patients at high ischemic and/or bleeding risk after percutaneous coronary intervention (PCI). Aspirin withdrawal within 3 months (Early) reduces bleeding without increasing myocardial infarction (MI), whereas Immediate noninitiation or cessation shows increases in early ischemic events. Forest plot of hazard ratios (HRs) with 95% confidence intervals. Pooled estimates were obtained using a random-effects model. *P* values for the overall pooled effect are from a two-sided *z* (Wald) test. Panel A colors indicate statistical significance (green: *p* < 0.05; blue: *p* ≥ 0.05). Panel B colors indicate timing strata (red: Immediate; blue: Early [within 3 months]). *N* = sample size.

Finally, to summarize the timing-dependent effects of aspirin withdrawal, a dedicated summary figure is provided (Fig 6), showing the overall pooled estimates for each outcome and the timing-stratified analysis comparing P2Y12 monotherapy (ticagrelor or prasugrel) versus DAPT. As shown in Fig 6, aspirin withdrawal within 3 months was associated with reduced bleeding without an increase in MI, whereas immediate noninitiation or cessation was associated with an excess of early ischemic events.

## Discussion

The findings of this large-scale meta-analysis of 27,743 high-risk patients undergoing PCI regarding aspirin discontinuation under potent P2Y12 monotherapy are at least 4-fold. First, compared with DAPT, aspirin discontinuation at ≤3 months significantly reduces major bleeding without an ischemic trade-off and without increasing mortality. Second, the timing of aspirin withdrawal critically determines safety: immediate (aspirin noninitiation or in-hospital cessation) increases MI, whereas discontinuation within 1–3 months preserves ischemic neutrality. Third, TSA confirmed conclusive evidence for bleeding benefit and futility for any MI advantage of prolonged DAPT. Fourth, for the first time Bayesian analyses quantified decision-relevant probabilities across risk-stratified scenarios, revealing risk-aligned timing: ≤1-month discontinuation for high bleeding risk (100% posterior probability of bleeding benefit; NNT = 12; 70% probability of MI-safety) and 3-month discontinuation for high ischemic risk (100% probability of bleeding benefit; NNT = 57; 86% probability of MI-safety). In aggregate, these four observations outline a pragmatic, timing-aware antiplatelet strategy that maximizes safety without significantly sacrificing ischemic protection.

The majority of patients across the included trials of this meta-analysis presented with ACS. Contemporary guidelines continue to recommend 12 months of DAPT with preferred potent P2Y12 inhibitor therapy as the default strategy after ACS, with consideration of shorter courses in high bleeding risk patients [2] and growing acknowledgement of P2Y12-monotherapy pathways after an initial DAPT period. These recommendations reflect the evidence base available when written. Historically, the “12-month default” arose less from randomized tests of duration and more from demonstrations of composition (the benefit of adding a P2Y12 inhibitor to aspirin) a finding established amid first-generation stent concerns [21–23]; with contemporary drug-eluting stents, intravascular imaging, and high-potency P2Y12 inhibitors, the benefit-risk calculus increasingly favors shortened DAPT followed by P2Y12-only therapy. Recent evidence has also consistently shown that more potent P2Y12 inhibitors—such as ticagrelor or prasugrel—yield lower ischemic and mortality outcomes over clopidogrel in high-risk patients [24–26]. Contemporary randomized trials in high-risk PCI populations have evaluated aspirin noninitiation or early discontinuation [3,4,6,19], yet uncertainties persist regarding optimal timing and whether effects are clinically meaningful.

A prior individual-patient data meta-analysis showed that de-escalation to ticagrelor monotherapy after short-term DAPT preserved protection from death, MI, or stroke while reducing major bleeding compared with 12-month DAPT in an overall PCI population [27]. However, in that analysis patients with chronic coronary syndrome randomized to prolonged DAPT received aspirin combined with either clopidogrel or ticagrelor, and the population was not restricted a priori to high-risk features. At variance with previous analyses, in which risk profile and/or potent P2Y₁₂ monotherapy were explored only in subgroup analyses, our study preserves the original randomized comparisons, incorporates the most recent trial evidence, and uses potent P2Y₁₂-based DAPT in both arms. This design isolates the effect of very short-DAPT durations (≤3 months) with a potent P2Y₁₂ inhibitor and specifically addresses a distinct knowledge gap by focusing exclusively on high-risk post-PCI patients treated with different short-DAPT strategies and randomized timing of aspirin withdrawal. In addition, the Bayesian framework allows probabilistic, scenario-based comparisons of these very short regimens across contrasting bleeding and ischemic risk profiles, moving beyond a single average treatment effect towards more individualized therapeutic guidance.

In antithrombotic trials, composite or endpoints may obscure clinically relevant trade-offs when ischemic and bleeding effects move in opposite directions. In the specific context of early aspirin withdrawal on top of potent P2Y12 inhibition, the key clinical question is whether bleeding can be reduced without increasing MI. We therefore prespecified MI and clinically relevant bleeding as co-primary efficacy and safety endpoints, rather than a single composite MACE, to provide a more transparent and clinically actionable assessment of the benefit–risk balance. Our overall findings—and those in pure ACS—indicate that, in high-risk patients, particularly ACS patients treated with contemporary drug-eluting stents and PCI practice, aspirin can be stopped after ≤1–3 months—provided the very early high ischemic risk window has passed—with substantial bleeding reduction and no ischemic trade-off under potent P2Y12 inhibitor maintenance.

The marked timing-dependent safety pattern of aspirin discontinuation observed in this meta-analysis reflects the pathophysiology of post-PCI thrombosis. Aspirin’s incremental anti-thromboxane effect is most consequential in the early post-PCI window, when ischemic events cluster after ACS within the first 30 days and stent thrombosis risk peaks [28]. As reendothelialization progresses and absolute ischemic hazard declines, aspirin’s bleeding liability persists without proportional ischemic benefit [29]. Thus, withdrawing aspirin within 3 months while maintaining potent P2Y12 inhibition unmasks a hemorrhagic advantage without ischemic compromise, whereas stopping too early may curtail protection before thrombotic risk has receded—consistent with early MI signals observed in immediate discontinuation studies [6,18]. Clinically, this physiology-to-outcome concordance provides a clear operational rule: avoid aspirin-free strategies in the first days after PCI, then plan discontinuation within 3 months once beyond the peak thrombotic phase, tailoring precise timing to individual bleeding and ischemic profiles. Beyond the first year after PCI, the choice between aspirin and P2Y₁₂-inhibitor monotherapy for long-term secondary prevention remains uncertain [30]: available mechanistic and trial data support sustained ADP–P2Y₁₂ pathway inhibition as a plausible [31], potentially bleeding-sparing option, but robust randomized evidence beyond 1–3 years and definitive comparative data versus aspirin are still lacking [32].

A compelling aspect of the current work is that the robust analytical approach applied strengthens inference through complementary methodologies. TSA demonstrates conclusiveness for bleeding benefit and futility for any MI advantage of prolonged DAPT, indicating further large trials are unlikely to alter these conclusions. The Bayesian framework converted treatment effects into decision-ready probabilities—namely, the probability of bleeding reduction (HR < 1.0) and the probability that MI risk remains within predefined limits (HR < 1.15)—and quantified these across prespecified risk-stratified scenarios. In high-bleeding-risk patients, ≤1-month aspirin discontinuation yielded a 100% posterior probability of bleeding benefit (NNT = 12) with moderate MI-safety confidence (70% probability of MI-safety). In high ischemic risk patients, ≤1-month discontinuation showed limited MI-safety confidence (20% probability), whereas 3-month discontinuation achieved both robust bleeding reduction (NNT = 57) and high MI-safety confidence (86% probability). These risk-stratified probabilistic estimates provide a quantitative framework for individualized decision-making in clinical practice. MI was not the primary endpoint in most included RCTs; therefore, MI analyses may be underpowered and more susceptible to chance findings and should be interpreted cautiously—although this concern is mitigated by our TSA supporting futility for detecting a clinically meaningful MI difference with additional trials. Overall, MI findings warrant confirmation in future adequately powered studies. Because composite MACE definitions were heterogeneous across studies, MI represents a clinically relevant and more consistently defined ischemic endpoint for cross-trial comparison.

The current landmark meta-analysis applying robust statistical methods supports a paradigm shift in the antithrombotic management of these patients. The current study—in patients stabilized on ticagrelor or prasugrel—supports a shortened-duration, timing-aware pathway in which aspirin is withdrawn within 3-months, delivering a clinically meaningful bleeding reduction without sacrificing MI or stroke occurrence.

Taken together, these observations suggest that early aspirin discontinuation under maintenance therapy with a potent P2Y12 inhibitor is safe in high-risk patients undergoing PCI. Clinicians may proactively plan aspirin cessation within a 3-month window after PCI, individualizing timing according to each patient’s bleeding and ischemic risk phenotype. Based on these results, future studies explicitly comparing alternative early stop-dates and P2Y12 inhibitor dose-de-escalation strategies may further refine clinical practice [31].

This meta-analysis synthesizes trial-level rather than individual-participant data, limiting granularity for specific high-risk phenotypes and endpoint harmonization. We mitigated this with prespecified timing strata, restriction to potent P2Y12 monotherapy under contemporary DES, and complementary TSA and Bayesian analyses. All main and sensitivity analyses confirmed the overall findings, suggesting that the effect is justified. Clopidogrel-based monotherapy was excluded by design because of its less favorable pharmacodynamic profile versus ticagrelor/prasugrel; while this narrows generalizability to clopidogrel, it enhances biological coherence and analytic homogeneity of the pooled estimates. Variation across regions and ethnicities (with several trials from East Asia) may limit generalizability to underrepresented populations. Nevertheless, effects were directionally consistent across prespecified timing and ACS-restricted analyses, confirmed by trial sequential and Bayesian models, and unchanged in leave-one-out analyses (Fig Q in S1 Appendix). Alternative random-effects models based on the Paule–Mandel estimator with Hartung–Knapp adjustment provided comparable results (Table E in S1 Appendix). Although our trial-selection criteria preserved randomization rather than relying on post-hoc subgroup analyses, we acknowledge that only two contributing trials employed an immediate aspirin withdrawal strategy. Accordingly, the analysis of the precise timing of aspirin withdrawal should be considered hypothesis-generating.

Among high-risk post-PCI patients on potent P2Y12 therapy, discontinuing aspirin at ≤1–3 months reduces bleeding without excess ischemic events, whereas immediate in-hospital noninitiation or cessation increases MI. Bayesian analyses support risk-aligned timing— ≤ 1 month for high bleeding risk (NNT for bleeding = 12) and 3 months for high ischemic risk (NNT = 57; MI-safety probability 86%). These findings support reconsideration of uniform 12-month DAPT in selected high-risk patients treated with potent P2Y12 inhibitors.

## Supporting information

S1 PRISMA Checklist PRISMA checklist for reporting systematic reviews and meta-analyses. Reproduced from: Page MJ, McKenzie JE, Bossuyt PM, et al. *The PRISMA 2020 statement: an updated guideline for reporting systematic reviews*. PLoS Med. 2021;18(3):e1003583. https://doi.org/10.1371/journal.pmed.1003583. This work is licensed under CC BY 4.0. https://creativecommons.org/licenses/by/4.0/.
(DOCX)

S1 Appendix Supporting information. **Fig A**. PRISMA flow chart of the meta-analysis. **Fig B**. Funnel plots for publication bias assessment. Funnel plots for publication bias assessment. HR = hazard ratio. **Fig C**. Myocardial infarction by timing of early aspirin discontinuation (≤1 month and 3 months) versus DAPT. Point estimates are hazard ratios (HRs) with 95% confidence intervals (CIs); symbol sizes are proportional to inverse-variance weights; diamonds denote pooled effects. **Fig D**. MI with potent P2Y12-inhibitor monotherapy versus DAPT in ACS. Point estimates are hazard ratios (HRs) with 95% confidence intervals (CIs); symbol sizes are proportional to inverse-variance weights; diamonds denote pooled effects. **Fig E**. Bleeding by timing of early aspirin discontinuation (≤1 month and 3 months) versus DAPT. Point estimates are hazard ratios (HRs) with 95% confidence intervals (CIs); symbol sizes are proportional to inverse-variance weights; diamonds denote pooled effects. **Fig F**. Bleeding with potent P2Y12-inhibitor monotherapy versus DAPT in ACS. Point estimates are hazard ratios (HRs) with 95% confidence intervals (CIs); symbol sizes are proportional to inverse-variance weights; diamonds denote pooled effects. **Fig G**. Major bleeding with potent P2Y12-inhibitor monotherapy versus DAPT. Point estimates are hazard ratios (HRs) with 95% confidence intervals (CIs); symbol sizes are proportional to inverse-variance weights; diamonds denote pooled effects. **Fig H**. Bayesian meta-analysis of MI and bleeding. Point estimates are hazard ratios (HRs) with 95% credible intervals (CrIs); symbol sizes are proportional to inverse-variance weights; diamonds denote pooled effects. **Fig I**. Death by timing of early aspirin discontinuation (immediate and early) versus DAPT. Point estimates are hazard ratios (HRs) with 95% confidence intervals (CIs); symbol sizes are proportional to inverse-variance weights; diamonds denote pooled effects. **Fig J**. Death by timing of early aspirin discontinuation (≤1 month and 3 months) versus DAPT. Point estimates are hazard ratios (HRs) with 95% confidence intervals (CIs); symbol sizes are proportional to inverse-variance weights; diamonds denote pooled effects. **Fig K**. Death with potent P2Y12-inhibitor monotherapy versus DAPT in ACS. Point estimates are hazard ratios (HRs) with 95% confidence intervals (CIs); symbol sizes are proportional to inverse-variance weights; diamonds denote pooled effects. **Fig L**. CV death with potent P2Y12-inhibitor monotherapy versus DAPT. Point estimates are hazard ratios (HRs) with 95% confidence intervals (CIs); symbol sizes are proportional to inverse-variance weights; diamonds denote pooled effects. **Fig M**. Stroke by timing of early aspirin discontinuation (immediate and early) versus DAPT. Point estimates are hazard ratios (HRs) with 95% confidence intervals (CIs); symbol sizes are proportional to inverse-variance weights; diamonds denote pooled effects. **Fig N**. Stroke by timing of early aspirin discontinuation (≤1 month and 3 months) versus DAPT. Point estimates are hazard ratios (HRs) with 95% confidence intervals (CIs); symbol sizes are proportional to inverse-variance weights; diamonds denote pooled effects. **Fig O**. Stroke with potent P2Y12-inhibitor monotherapy versus DAPT in ACS. Point estimates are hazard ratios (HRs) with 95% confidence intervals (CIs); symbol sizes are proportional to inverse-variance weights; diamonds denote pooled effects. **Fig P**. Definite/probable stent thrombosis with potent P2Y12-inhibitor monotherapy versus DAPT. Point estimates are hazard ratios (HRs) with 95% confidence intervals (CIs); symbol sizes are proportional to inverse-variance weights; diamonds denote pooled effects. **Fig Q**. Leave-one-out sensitivity analysis. Point estimates are hazard ratios (HRs) with 95% confidence intervals (CIs); symbol sizes are proportional to inverse-variance weights; diamonds denote pooled effects. **Table A**. Myocardial infarction definitions across trials. MI = myocardial infarction. **Table B**. Risk of Bias (RoB 2)—Aspirin Discontinuation Trials. Cochrane RoB 2 domains: D1 Randomization process; D2 Deviations from intended interventions (effect of assignment); D3 Missing outcome data; D4 Measurement of the outcome; D5 Selection of the reported result. Overall judgement per study. **Table C**. The certainty of evidence (GRADE) for investigated outcomes. **Table D**. Posterior Bayesian samples. HR = hazard ratio. Cri = credible interval. The τ symbol denotes between-trial variance. **Table E**. Random-effects models based on the Paule–Mandel estimator with Hartung–Knapp adjustment. HR = hazard ratio. CI = confidence interval.
(DOCX)

## Abbreviations

ACS: acute coronary syndrome
ARC-HBR: Academic Research Consortium high bleeding risk
BARC: Bleeding Academic Research Consortium
CI: confidence interval
CrI: credible interval
DAPT: dual antiplatelet therapy
DES: drug eluting stent(s)
HR: hazard ratio
MI: myocardial infarction
NNT: number needed to treat
PCI: percutaneous coronary intervention
RIS: required information size
TSA: trial sequential analysis.
