# Supplementary material for: Early aspirin withdrawal versus dual antiplatelet therapy in high-risk patients after percutaneous coronary intervention: Meta-analysis of randomized trials
Source: PLoS Med. 2026 Mar 26;23(3):e1004995. doi: 10.1371/journal.pmed.1004995 (PMC13020786; doi:10.1371/journal.pmed.1004995)
Supplement: S1 Appendix — Fig A. PRISMA flow chart of the meta-analysis. Fig B. Funnel plots for publication bias assessment. Funnel plots for publication bias assessment. HR = hazard ratio. Fig C. Myocardial infarction by timing of early aspirin discontinuation (≤1 month and 3 months) versus DAPT. Point estimates are hazard ratios (HRs) with 95% confidence intervals (CIs); symbol sizes are proportional to inverse-variance weights; diamonds denote pooled effects. Fig D. MI with potent P2Y12-inhibitor monotherapy versus DAPT in ACS. Point estimates are hazard ratios (HRs) with 95% confidence intervals (CIs); symbol sizes are proportional to inverse-variance weights; diamonds denote pooled effects. Fig E. Bleeding by timing of early aspirin discontinuation (≤1 month and 3 months) versus DAPT. Point estimates are hazard ratios (HRs) with 95% confidence intervals (CIs); symbol sizes are proportional to inverse-variance weights; diamonds denote pooled effects. Fig F. Bleeding with potent P2Y12-inhibitor monotherapy versus DAPT in ACS. Point estimates are hazard ratios (HRs) with 95% confidence intervals (CIs); symbol sizes are proportional to inverse-variance weights; diamonds denote pooled effects. Fig G. Major bleeding with potent P2Y12-inhibitor monotherapy versus DAPT. Point estimates are hazard ratios (HRs) with 95% confidence intervals (CIs); symbol sizes are proportional to inverse-variance weights; diamonds denote pooled effects. Fig H. Bayesian meta-analysis of MI and bleeding. Point estimates are hazard ratios (HRs) with 95% credible intervals (CrIs); symbol sizes are proportional to inverse-variance weights; diamonds denote pooled effects. Fig I. Death by timing of early aspirin discontinuation (immediate and early) versus DAPT. Point estimates are hazard ratios (HRs) with 95% confidence intervals (CIs); symbol sizes are proportional to inverse-variance weights; diamonds denote pooled effects. Fig J. Death by timing of early aspirin discontinuation (≤1 month and 3 months) versus DAPT. [file pmed.1004995.s002.docx]

**Early Aspirin withdrawal vs DAPT in High-Risk Patients undergoing PCI: Meta-analysis of randomized trials**

Navarese et al.

[**Figure A. PRISMA flow chart** 2](#_Toc223698771)

[**Figure B. Funnel plots for publication bias assessment.** 2](#_Toc223698772)

[**Figure C. Myocardial infarction by timing of early aspirin discontinuation (≤1 month and 3 months) versus DAPT** 4](#_Toc223698773)

[**Figure D. MI with potent P2Y12-inhibitor monotherapy versus DAPT in ACS.** 4](#_Toc223698774)

[**Figure E. Bleeding by timing of early aspirin discontinuation (≤1 month and 3 months) versus DAPT** 4](#_Toc223698775)

[**Figure F. Bleeding with potent P2Y12-inhibitor monotherapy versus DAPT in ACS.** 5](#_Toc223698776)

[**Figure G. Major bleeding with potent P2Y12-inhibitor monotherapy versus DAPT** 5](#_Toc223698777)

[**Figure H. Bayesian meta-analysis of MI and bleeding.** 6](#_Toc223698778)

[**Figure I. Death by timing of early aspirin discontinuation (immediate and early) versus DAPT** 7](#_Toc223698779)

[**Figure J. Death by timing of early aspirin discontinuation (≤1 month and 3 months) versus DAPT** 7](#_Toc223698780)

[**Figure K. Death with potent P2Y12-inhibitor monotherapy versus DAPT in ACS.** 8](#_Toc223698781)

[**Figure L. CV death with potent P2Y12-inhibitor monotherapy versus DAPT** 8](#_Toc223698782)

[**Figure M. Stroke by timing of early aspirin discontinuation (immediate and early) versus DAPT** 9](#_Toc223698783)

[**Figure N. Stroke by timing of early aspirin discontinuation (≤1 month and 3 months) versus DAPT** 10](#_Toc223698784)

[**Figure O. Stroke with potent P2Y12-inhibitor monotherapy versus DAPT in ACS.** 10](#_Toc223698785)

[**Figure P. Definite/probable stent thrombosis with potent P2Y12-inhibitor monotherapy versus DAPT** 11](#_Toc223698786)

[**Figure Q. Leave-one-out sensitivity analysis** 11](#_Toc223698787)

[**Table A.** Myocardial infarction definitions across trials**.** 11](#_Toc223698788)

[**Table B.** Risk of Bias (RoB 2) — Aspirin Discontinuation Trials 12](#_Toc223698789)

[**Table C.** The certainty of evidence (GRADE). 14](#_Toc223698790)

[**Table D.** Posterior bayesian samples 14](#_Toc223698791)

[**Table E.** Random-effects models based on the Paule–Mandel estimator with Hartung–Knapp adjustment 14](#_Toc223698792)

# **Figure A. PRISMA flow chart**


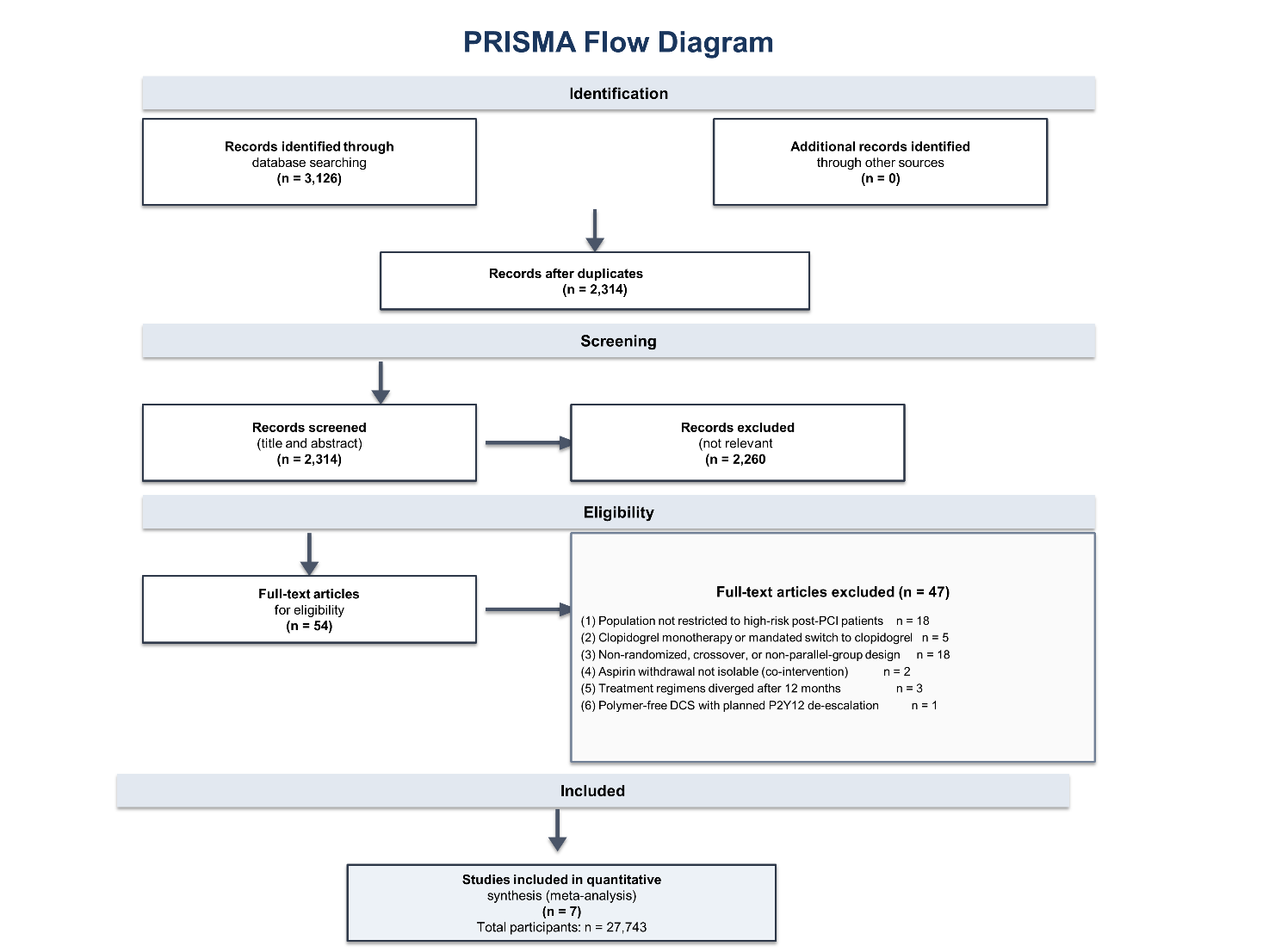


# **Figure B. Funnel plots for publication bias assessment.**


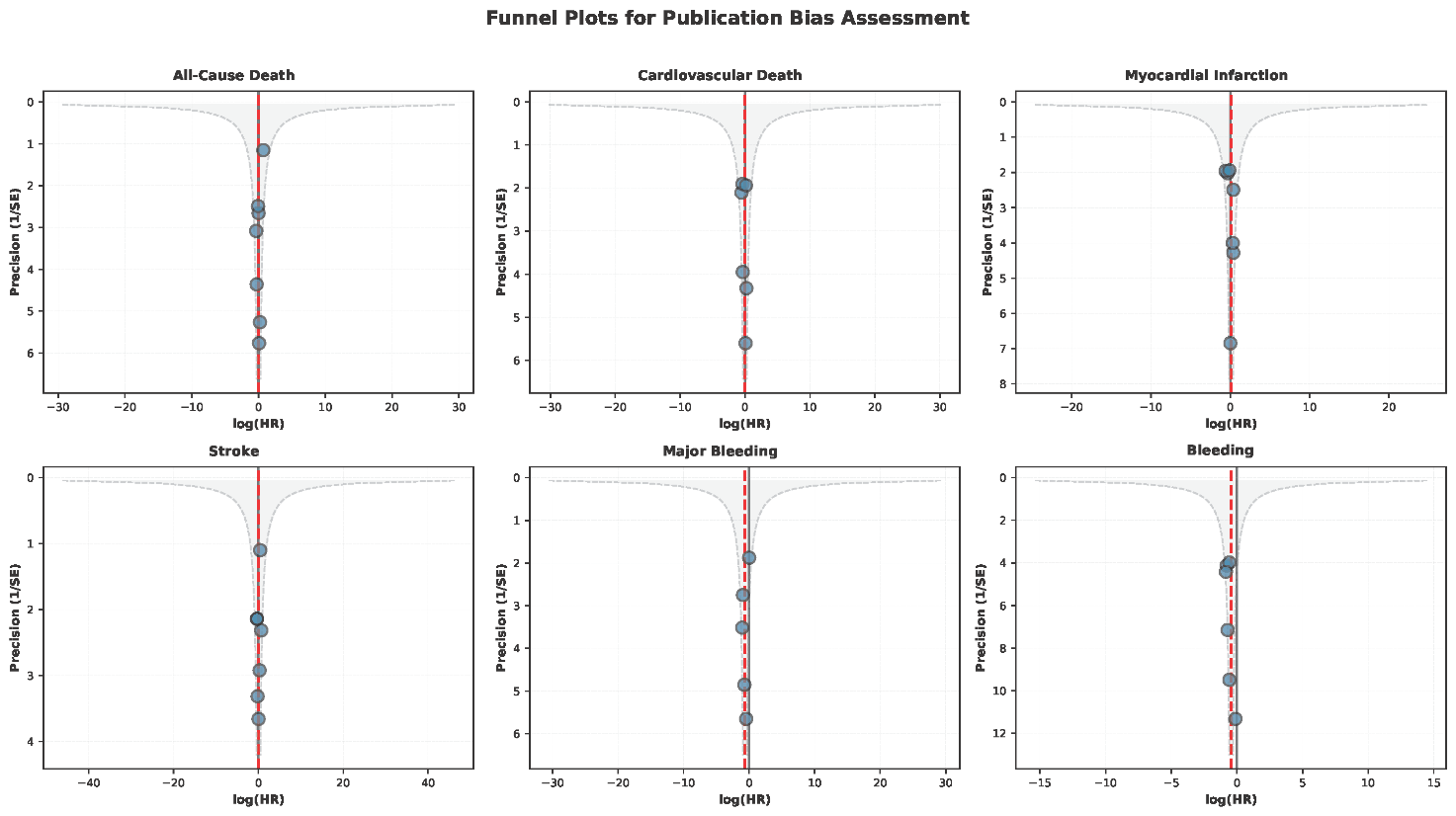


# **Figure C. Myocardial infarction by timing of early aspirin discontinuation (≤1 month and 3 months) versus DAPT**


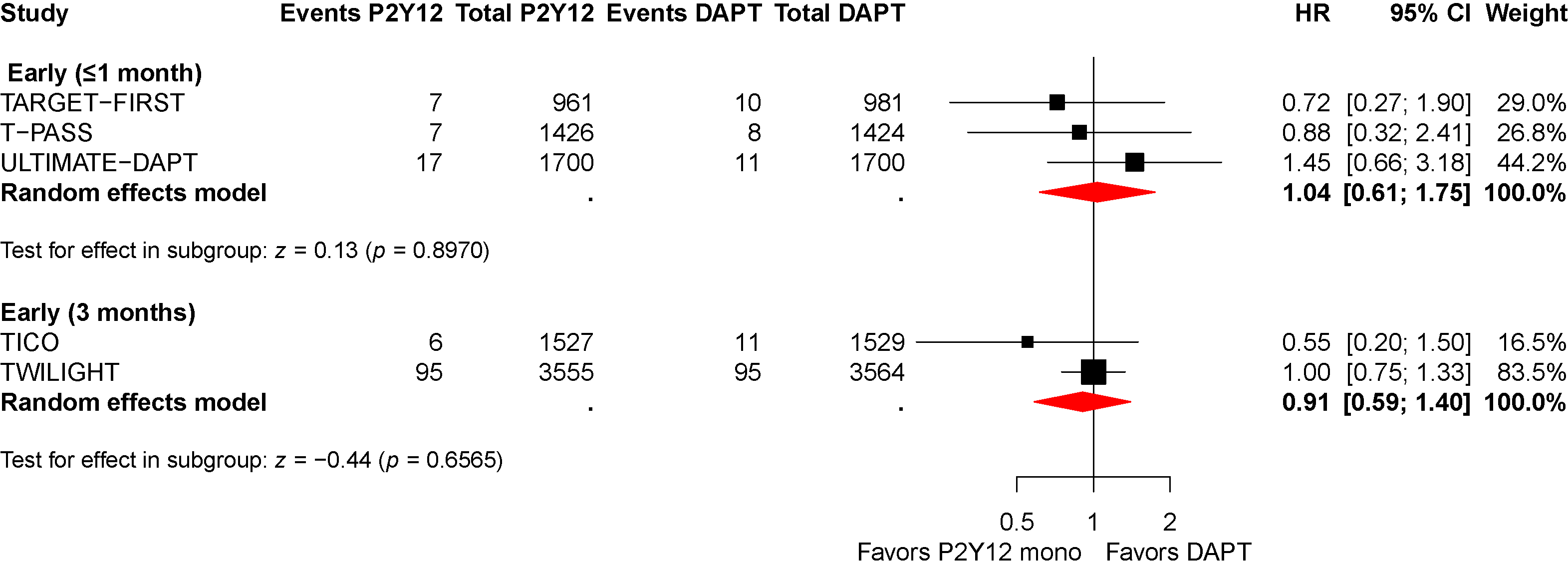


# **Figure D. MI with potent P2Y12-inhibitor monotherapy versus DAPT in ACS.**


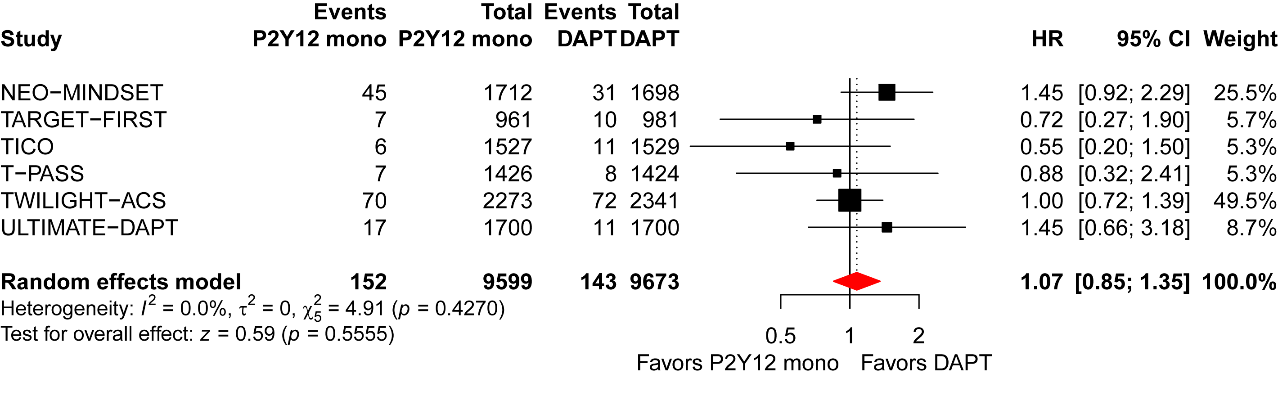


# **Figure E. Bleeding by timing of early aspirin discontinuation (≤1 month and 3 months) versus DAPT**


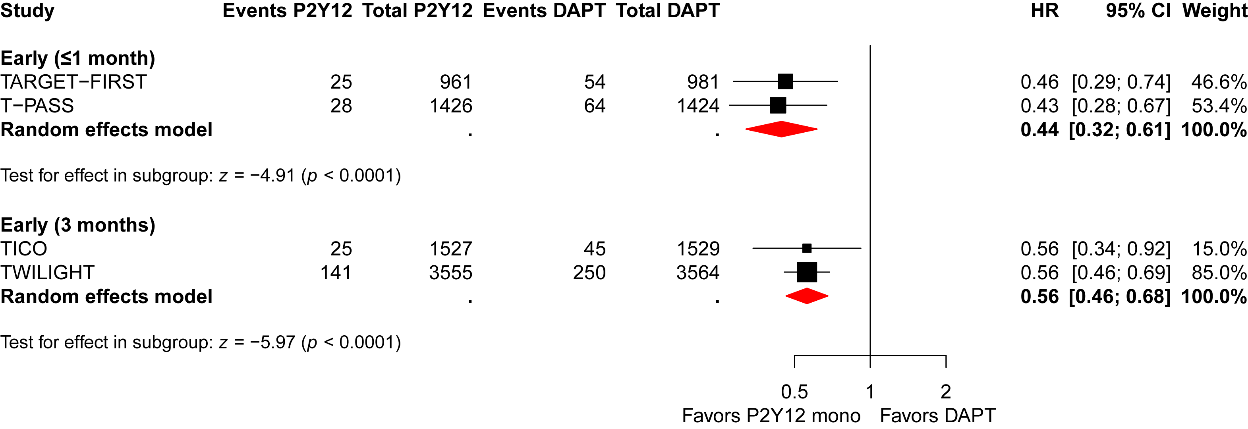


# **Figure F. Bleeding with potent P2Y12-inhibitor monotherapy versus DAPT in ACS.**


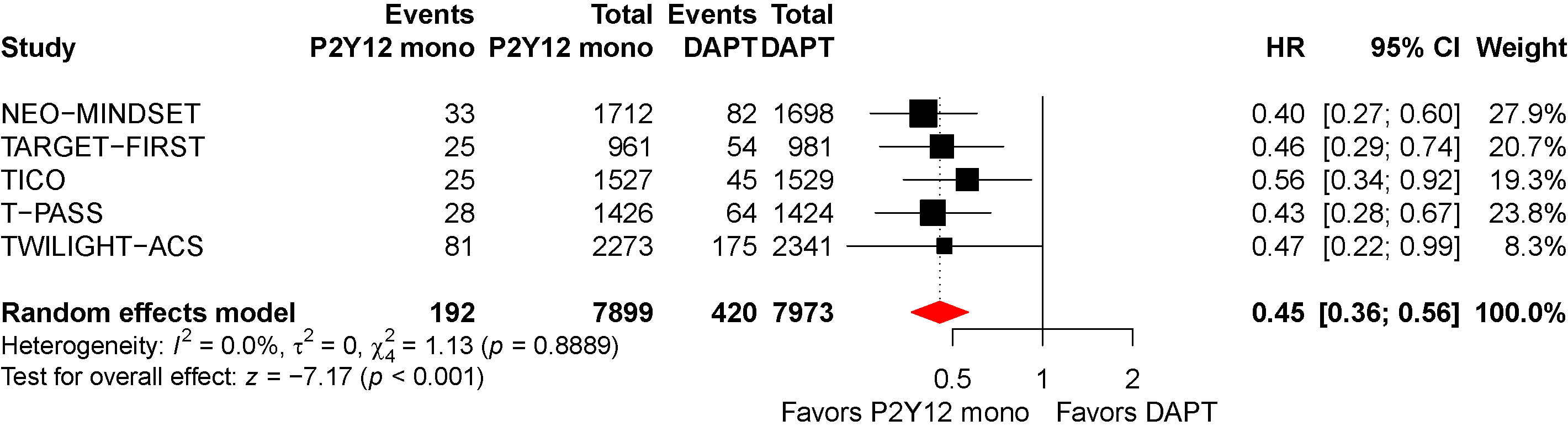


# **Figure G. Major bleeding with potent P2Y12-inhibitor monotherapy versus DAPT**


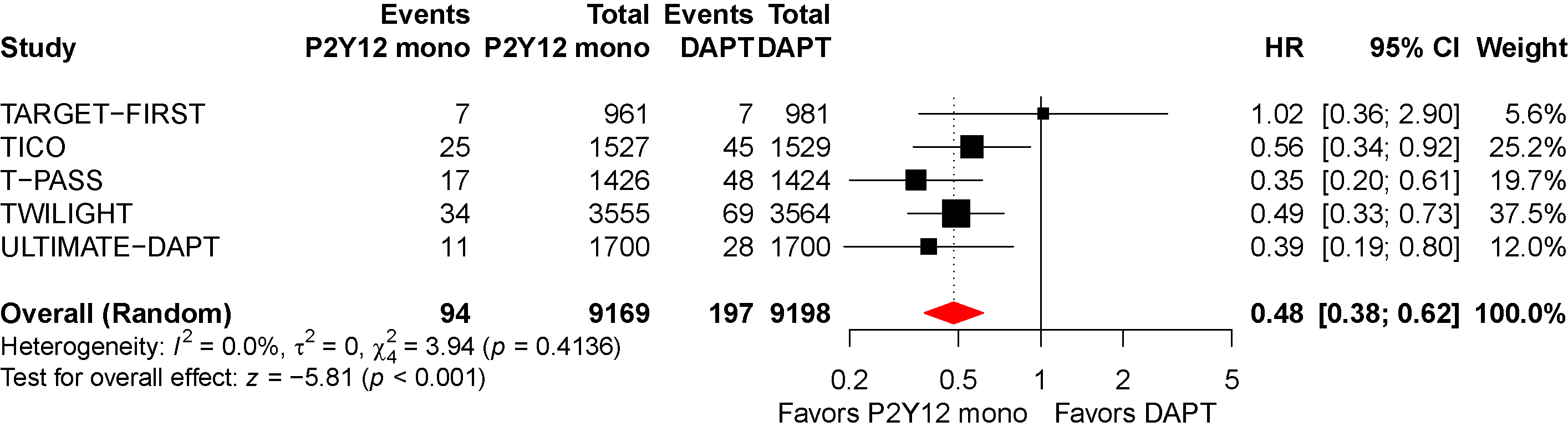


# **Figure H. Bayesian meta-analysis of MI and bleeding.**


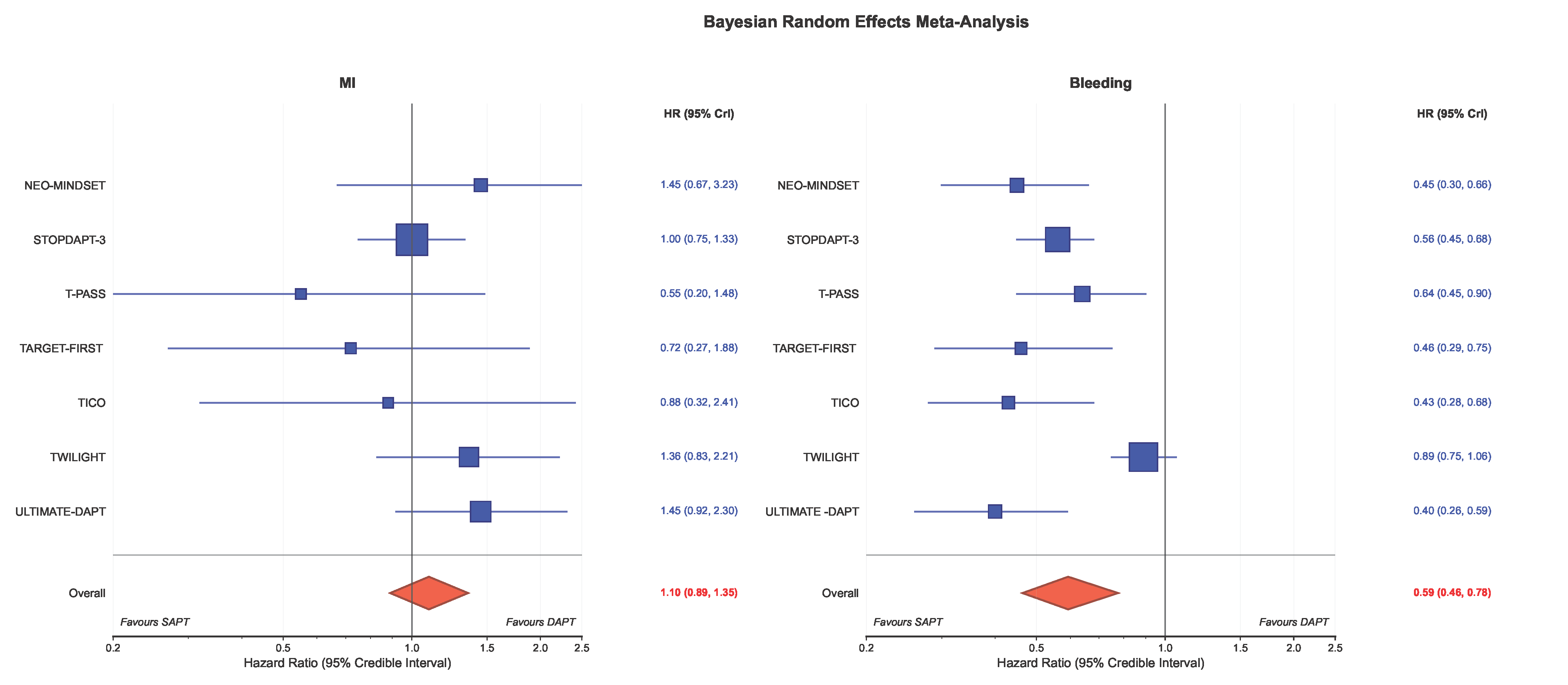


# **Figure I. Death by timing of early aspirin discontinuation (immediate and early) versus DAPT**


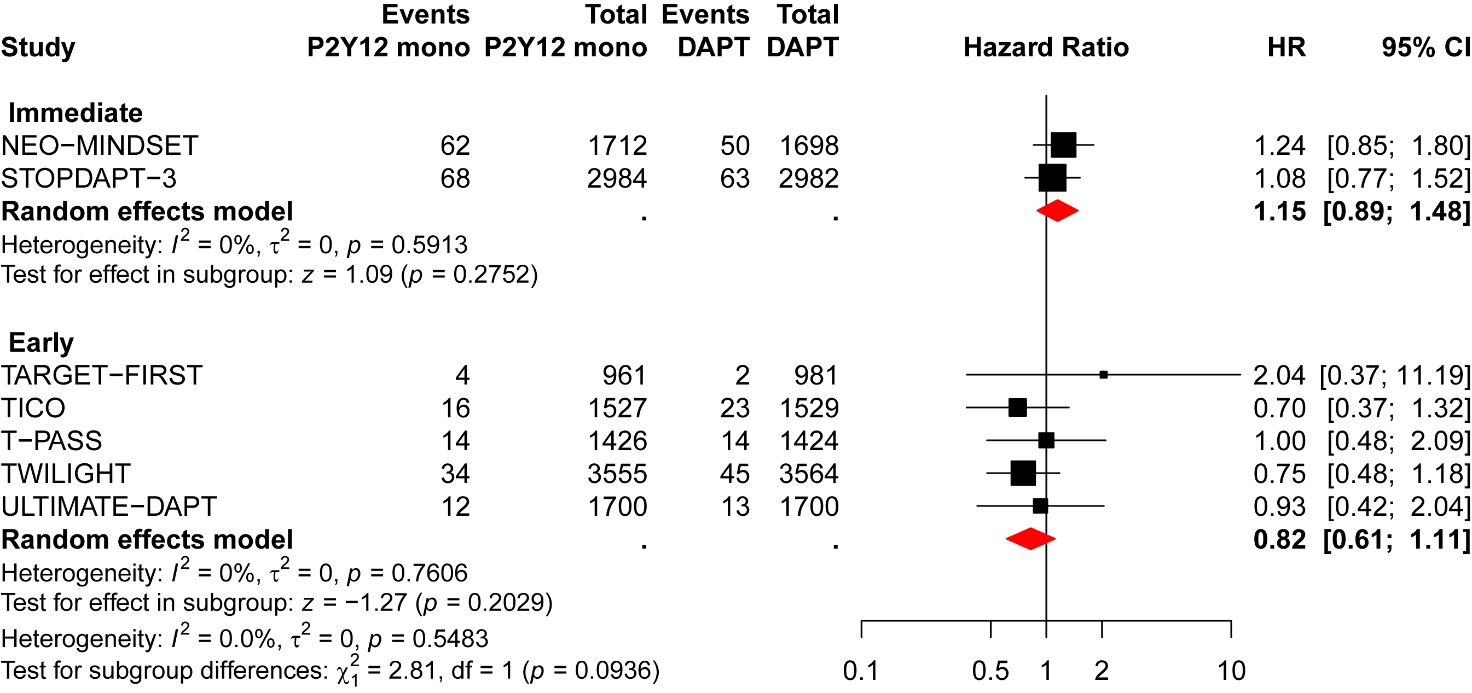


# **Figure J. Death by timing of early aspirin discontinuation (≤1 month and 3 months) versus DAPT**


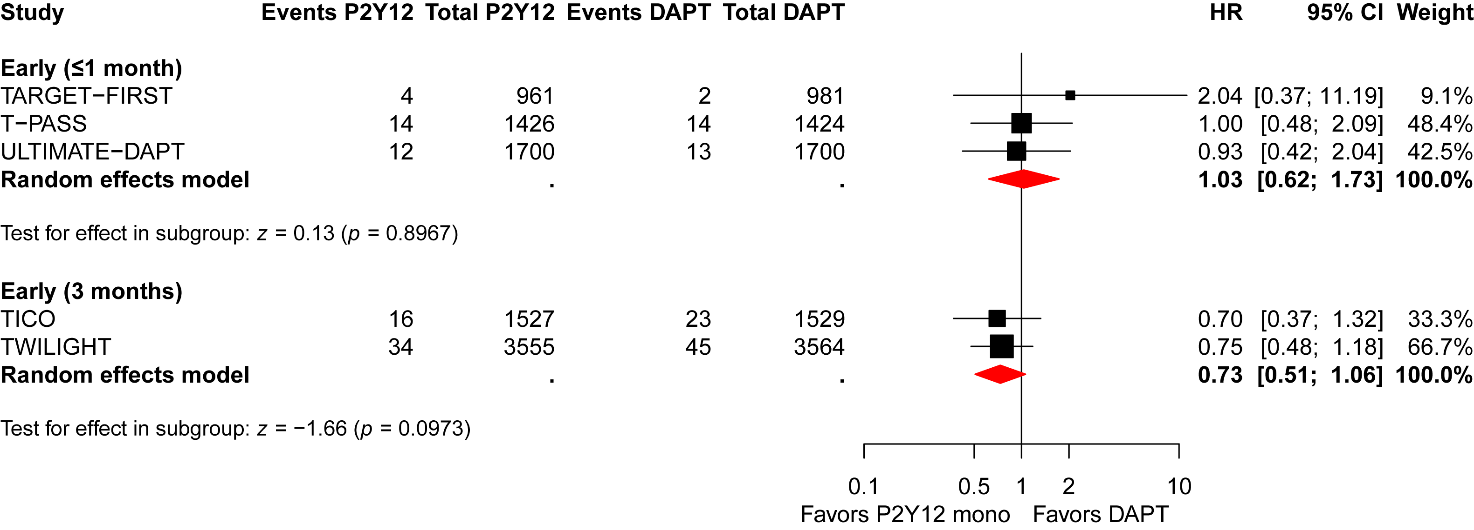


# **Figure K. Death with potent P2Y12-inhibitor monotherapy versus DAPT in ACS.**


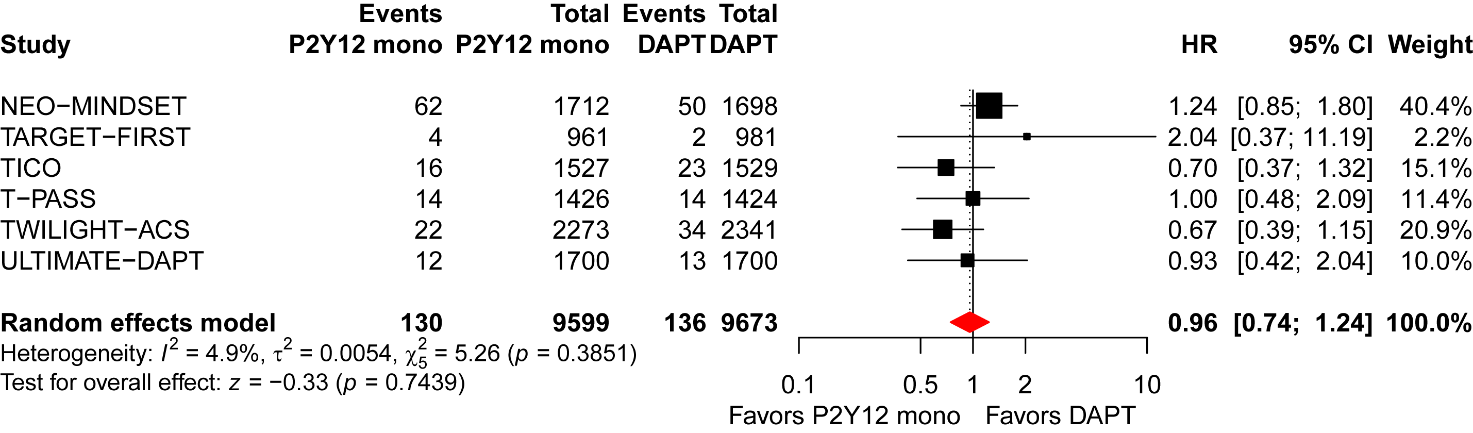


# **Figure L. CV death with potent P2Y12-inhibitor monotherapy versus DAPT**


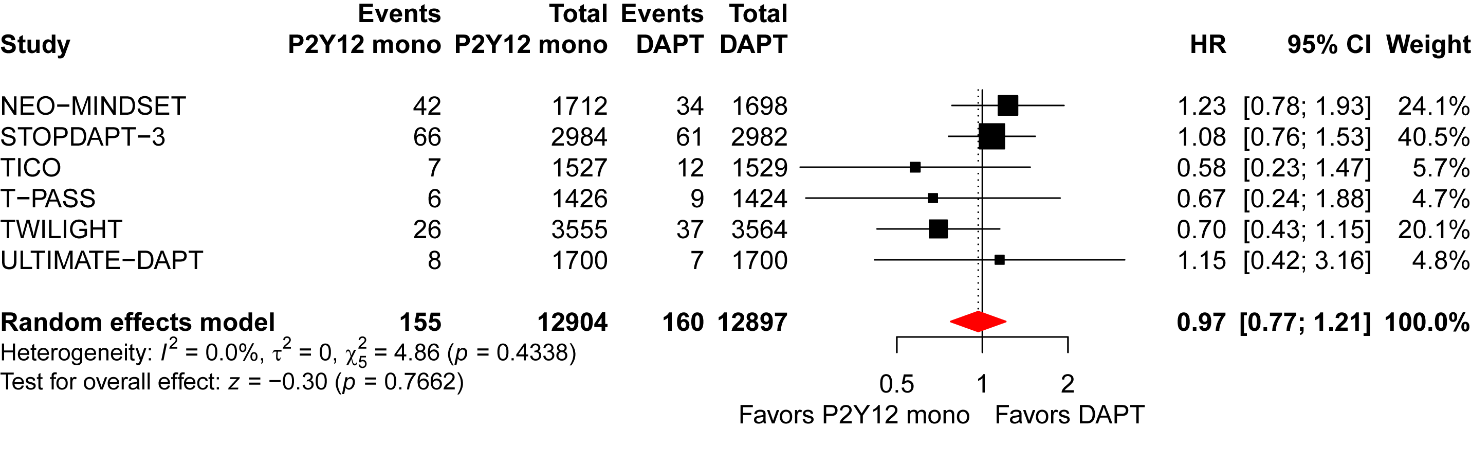


# **Figure M. Stroke by timing of early aspirin discontinuation (immediate and early) versus DAPT**


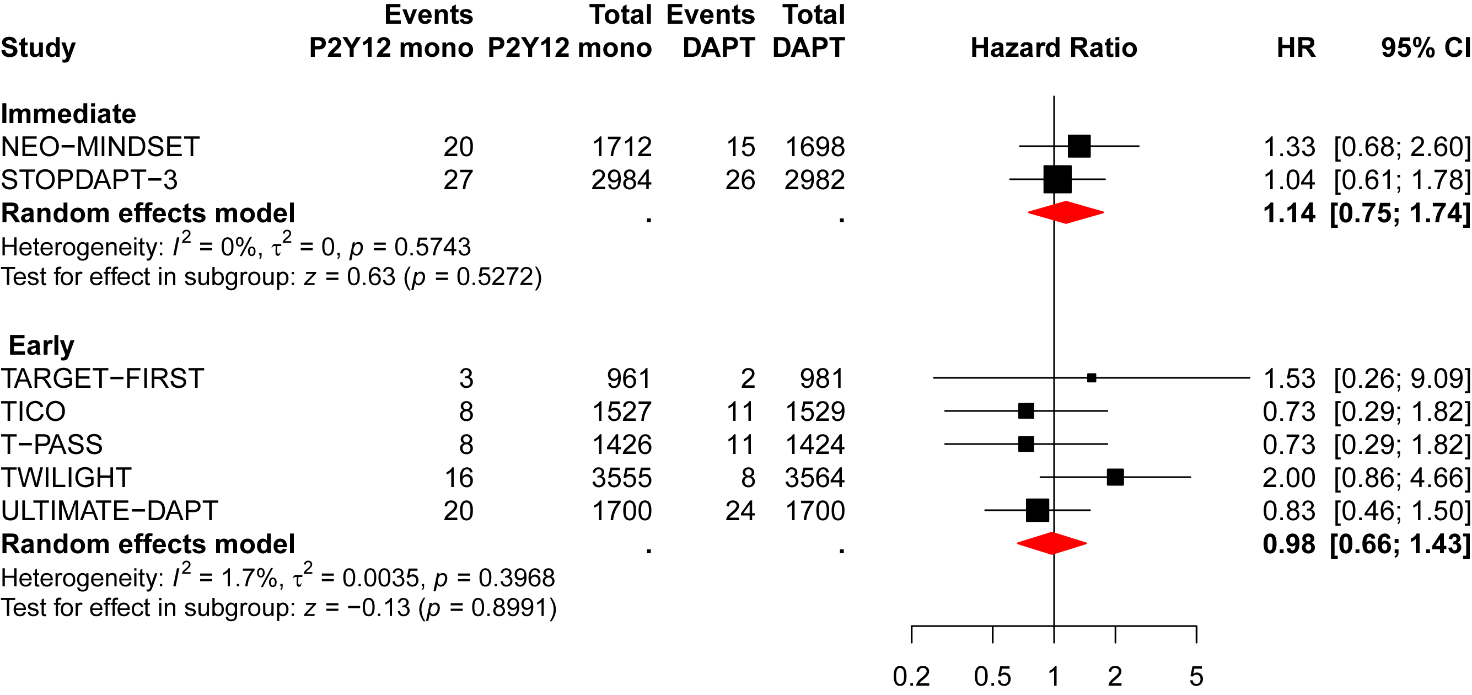


# **Figure N. Stroke by timing of early aspirin discontinuation (≤1 month and 3 months) versus DAPT**


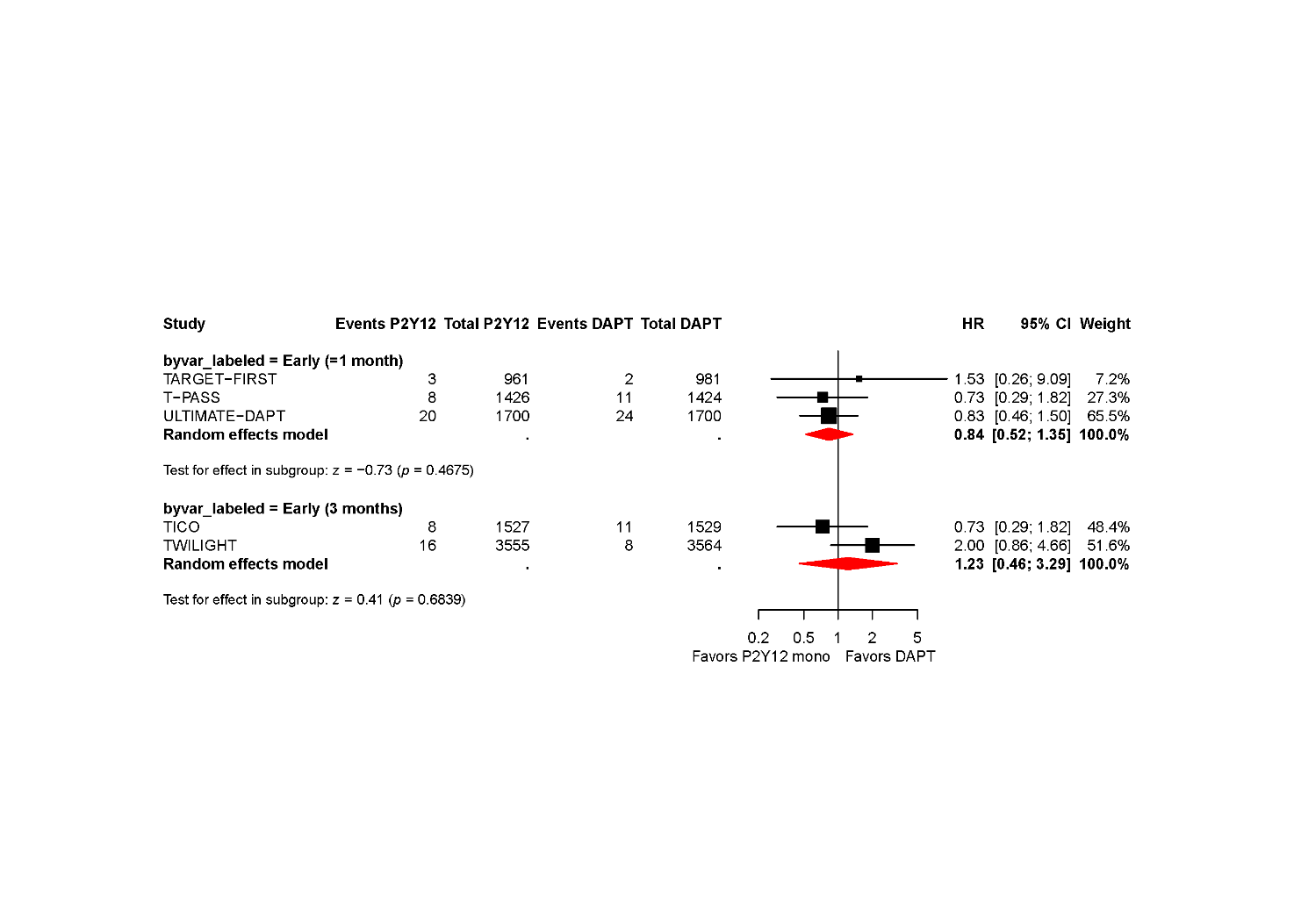


# **Figure O. Stroke with potent P2Y12-inhibitor monotherapy versus DAPT in ACS.**


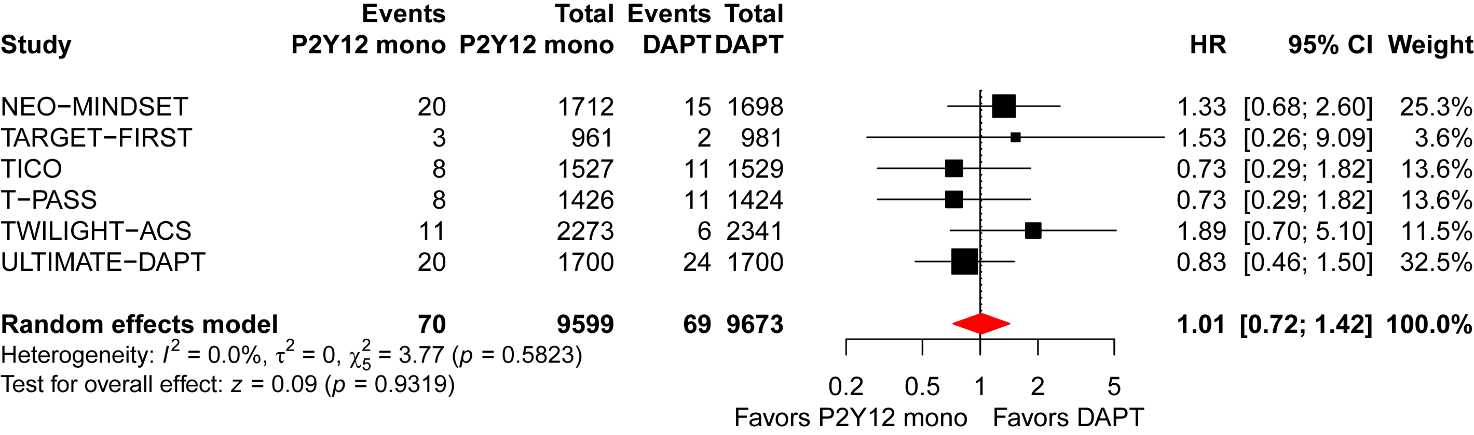


# **Figure P. Definite/probable stent thrombosis with potent P2Y12-inhibitor monotherapy versus DAPT**


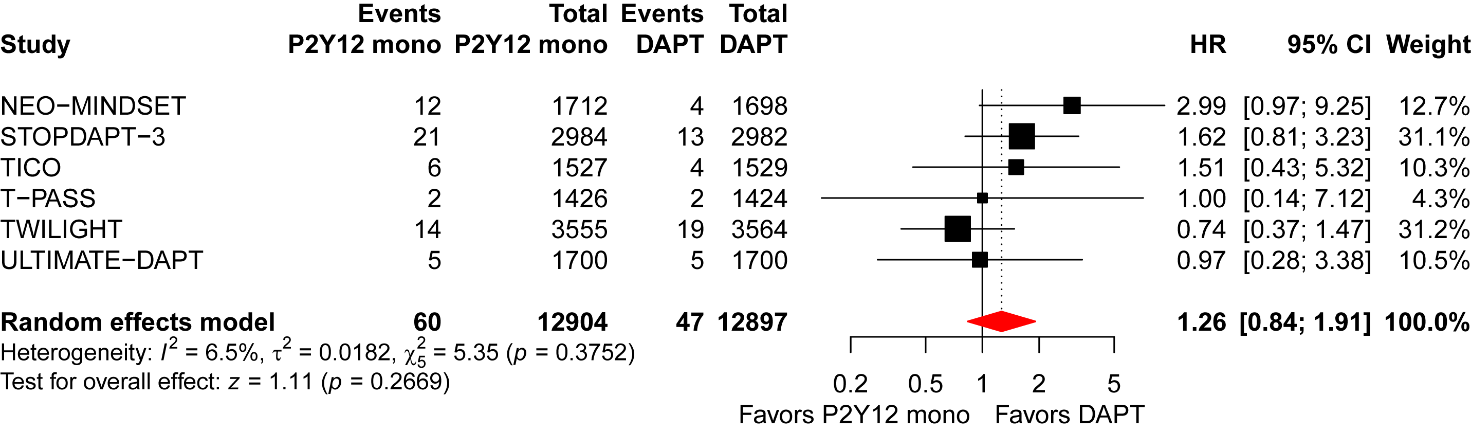


# **Figure Q. Leave-one-out sensitivity analysis**


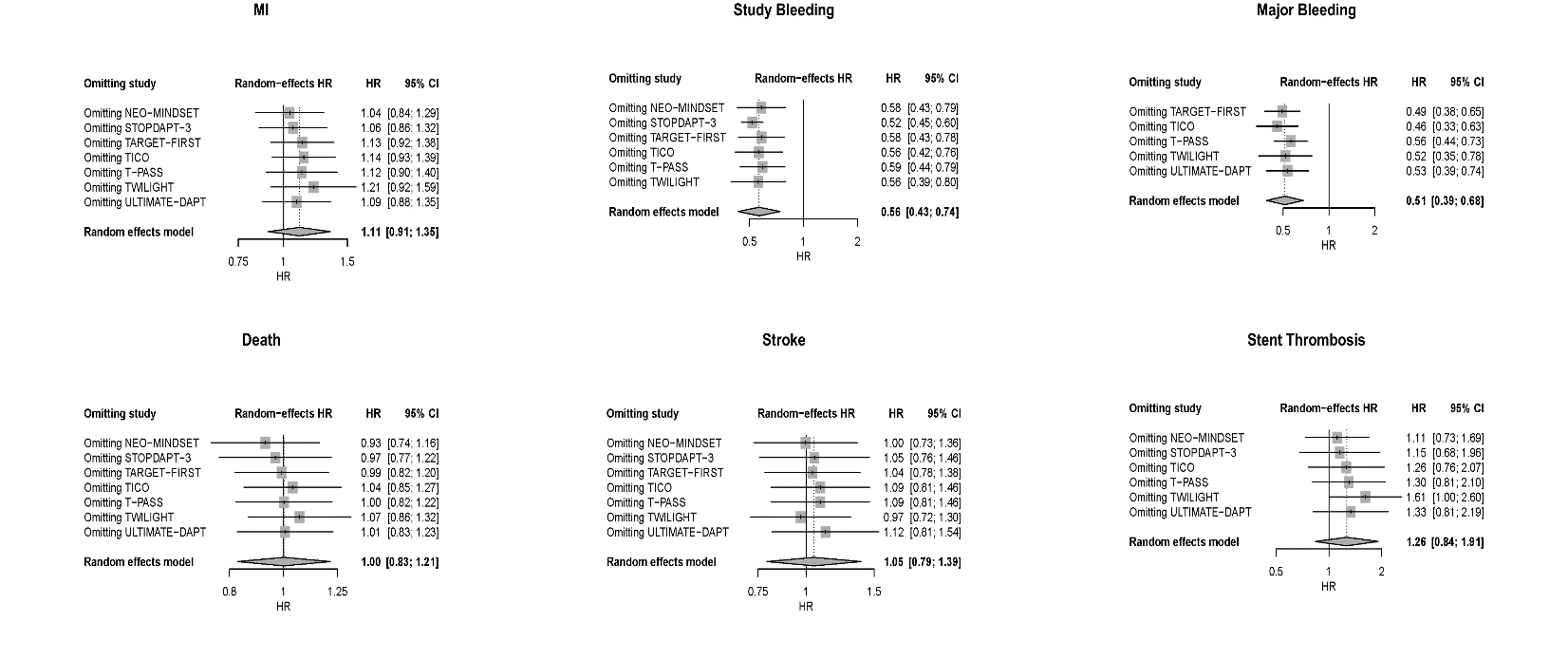


# **Table A.** Myocardial infarction definitions across trials**.**

| Trial | Year | MI Definition |
| --- | --- | --- |
| TWILIGHT | 2019 | Third Universal Definition of MI |
| TICO | 2020 | Third Universal Definition of MI |
| STOPDAPT-3 | 2023 | Academic ResearchConsortium (ARC) criteria |
| T-PASS | 2023 | Universal Definition of MI* |
| ULTIMATE-DAPT | 2022 | Third Universal Definition of MI |
| NEO-MINDSET | 2024 | Fourth Universal Definition of MI |
| TARGET-FIRST | 2024 | Fourth Universal Definition of MI |

*MI definition not explicitly stated in the primary publication but consistent withcontemporary universal definition criteria; all events adjudicated by independent clinicalendpoint committees.

# **Table B.** Risk of Bias (RoB 2) — Aspirin Discontinuation Trials

Cochrane RoB 2 domains: D1 Randomization process; D2 Deviations from intended interventions (effect of assignment); D3 Missing outcome data; D4 Measurement of the outcome; D5 Selection of the reported result. Overall judgement per study.

| Study | D1: Randomization | D2: Deviations (assignment) | D3: Missing data | D4: Outcome measurement | D5: Reporting selection | Overall risk | Notes (justification) |
| --- | --- | --- | --- | --- | --- | --- | --- |
| TWILIGHT (2019) | Low | Low | Low | Low | Low | Low | Double-blind, placebo-controlled after 3 months DAPT; central adjudication; low attrition. |
| ULTIMATE-DAPT (2024) | Low | Low | Low | Low | Low | Low | Randomized, double-blind at 1 month; blinded outcome assessment; minimal missing data. |
| NEO-MINDSET (2024) | Low | Some concerns | Low | Some concerns | Low | Some concerns | Open-label immediate aspirin withdrawal; objective outcomes but bleeding ascertainment may be influenced by knowledge of allocation. |
| STOPDAPT-3 (2023) | Low | Some concerns | Low | Some concerns | Low | Some concerns | Open-label; co-primary outcomes at 1 month; potential performance/detection bias; low missing data. |
| TARGET-FIRST (2024) | Low | Some concerns | Low | Some concerns | Low | Some concerns | Open-label randomization at 1 month (event-free); clinical adjudication reported; risk of deviations from intended care. |
| TICO (2020) | Low | Some concerns | Low | Some concerns | Low | Some concerns | Open-label; outcomes centrally adjudicated; major bleeding objective (BARC 3/5) but knowledge of treatment may affect care. |
| T-PASS / Hong (2024) | Low | Some concerns | Low | Some concerns | Low | Some concerns | Open-label; multicenter; objective outcomes; low attrition; possible detection/performance bias. |

## **Table C.** The certainty of evidence (GRADE).

| **Outcome** | **Certainty of evidence (GRADE)** |
| --- | --- |
| Clinically relevant bleeding | **High** |
| Myocardial infarction (MI)* | **Moderate–High** |
| All-cause death | **High** |
| Stroke | **Moderate** |

* **MI note:** Overall certainty is **moderate–high** due to timing variations; excluding the “immediate” subgroup yields **high** certainty.

# **Table D.** Posterior bayesian samples

| **Endpoint** | **Posterior samples** | **Prior τ scale** | **HR (median)** | **95% CrI (HR)** | **P(HR < 1)** |
| --- | --- | --- | --- | --- | --- |
| Bleeding | 10,000 | 0.20 | 0.53 | 0.41–0.68 | 1.00 |
| Bleeding | 50,000 | 0.20 | 0.53 | 0.41–0.68 | 1.00 |
| Endpoint | Posterior samples | Prior τ scale | HR (median) | 95% CrI (HR) | P(HR > 1) |
| MI | 10,000 | 0.20 | 1.10 | 0.89–1.35 | 0.81 |
| MI | 50,000 | 0.20 | 1.10 | 0.89–1.35 | 0.81 |

# **Table E.** Random-effects models based on the Paule–Mandel estimator with Hartung–Knapp adjustment

| **Outcome** | **HR** | **CI_Lower** | **CI_Upper** | **P_Value** |
| --- | --- | --- | --- | --- |
| Cardiovascular Death | 0.97 | 0.73 | 1.29 | 0.775 |
| All-Cause Death | 1 | 0.81 | 1.25 | 0.9867 |
| Myocardial Infarction | 1.11 | 0.87 | 1.41 | 0.349 |
| Bleeding | 0.55 | 0.42 | 0.73 | 0.0019 |
| Stroke | 1.05 | 0.77 | 1.43 | 0.7265 |
| Major Bleeding | 0.48 | 0.34 | 0.68 | 0.0043 |
